# Mechanics of the brain: perspectives, challenges, and opportunities

**DOI:** 10.1007/s10237-015-0662-4

**Published:** 2015-02-26

**Authors:** Alain Goriely, Marc G. D. Geers, Gerhard A. Holzapfel, Jayaratnam Jayamohan, Antoine Jérusalem, Sivabal Sivaloganathan, Waney Squier, Johannes A. W. van Dommelen, Sarah Waters, Ellen Kuhl

**Affiliations:** 1 Mathematical Institute, University of Oxford, Oxford, OX2 6GG UK; 2 Materials Technology Institute, Eindhoven University of Technology, 5600 MB Eindhoven, The Netherlands; 3 Institute of Biomechanics, Graz University of Technology, 8010 Graz, Austria; 4 Department of Pediatric Neurosurgery, John Radcliffe Hospital, Oxford, OX3 9DU UK; 5 Department of Engineering Science, University of Oxford, Oxford, OX1 3JP UK; 6 Department of Applied Mathematics, University of Waterloo, Waterloo, ON N2L3G1 Canada; 7 Department of Neuropathology, John Radcliffe Hospital, Oxford, OX3 9DU UK; 8 Departments of Mechanical Engineering and Bioengineering, Stanford University, Stanford, CA 94305 USA

**Keywords:** Brain, Solid mechanics, Fluid mechanics, Electrochemistry, Electromechanics, Traumatic brain injury

## Abstract

The human brain is the continuous subject of extensive investigation aimed at understanding its behavior and function. Despite a clear evidence that mechanical factors play an important role in regulating brain activity, current research efforts focus mainly on the biochemical or electrophysiological activity of the brain. Here, we show that classical mechanical concepts including deformations, stretch, strain, strain rate, pressure, and stress play a crucial role in modulating both brain form and brain function. This opinion piece synthesizes expertise in applied mathematics, solid and fluid mechanics, biomechanics, experimentation, material sciences, neuropathology, and neurosurgery to address today’s open questions at the forefront of neuromechanics. We critically review the current literature and discuss challenges related to neurodevelopment, cerebral edema, lissencephaly, polymicrogyria, hydrocephaly, craniectomy, spinal cord injury, tumor growth, traumatic brain injury, and shaken baby syndrome. The multi-disciplinary analysis of these various phenomena and pathologies presents new opportunities and suggests that mechanical modeling is a central tool to bridge the scales by synthesizing information from the molecular via the cellular and tissue all the way to the organ level.

## Motivation

The human brain is an organ of extreme complexity, the object of ultimate intellectual egocentrism, and a source of endless scientific challenges. At the basic functional level, the goal of many scientific inquiries is to understand the functions that result from the interaction of about 86 billion neurons with 100 trillion connections. From this perspective, the problem consists of connecting the biochemical and electrophysiological behavior of brain cells with the overall behavior of networks of connected cells. The ultimate goal is to translate the resulting macroscopic electrophysiological behavior into the functional dimension where direct relations can be established with neuronal response and, ultimately, behavior.

Despite an overwhelming interest and major research initiatives on how our brain operates, comparatively little is known about how the brain functions at the mechanical level. Recent findings have directly linked major brain development, mechanisms, and diseases to the mechanical response of the brain both at the cellular and tissue levels. Various factors contribute to this poor state of knowledge. First, the brain is a fully enclosed organ that is particularly difficult to probe physically. Second, viewed as a solid, it is extremely soft and its mechanical response is heavily influenced by a fluid phase and multiple charged molecules found in its cells and in the extracellular matrix. A holistic mechanical analysis requires a fully coupled multi-field theory, which needs to be calibrated and validated experimentally. Further, most brain pathologies depend on many different factors and their physical manifestation may be conveniently ignored by focusing on genetics and cellular function as the primary driver. This apparent lack of interest from both the brain and mechanics communities is also in clear distinction with other major organs such as the heart, arteries, lungs, or bones for which there exist well-established theories and large scientific communities. Nonetheless, the last decade has seen fundamental advances in different areas of brain mechanics and has revealed that one of the reasons that brain mechanics is particularly exciting is that it involves extreme scales: the extremely soft scale associated with neurosurgery; the extremely hard scale associated with the skull; the extremely slow scale associated with brain development; and the extremely fast scale associated with traumatic brain injury.

The objective of this contribution is not to give an extensive, comprehensive review of brain mechanics. Rather, it is both a primer into the basic physical principles underlying brain function and a window into a number of problems and challenges that the authors found to be of current interest. In the first part of this manuscript, we present the current understanding of the fundamental mechanics of the brain by reviewing its solid, fluid, electrochemical, and electromechanical components. In the second part, we discuss a number of situations and pathologies where mechanics play a key role and where modeling can improve our understanding and predicting capabilities. These include brain development, brain tumors, brain surgery, traumatic brain injury, and shaken baby syndrome. Our aim is to motivate further research in this area and to argue for a global approach of brain mechanics linking molecular, cellular, tissue, and organ scales.

## Solid mechanics

We begin our overview by discussing the solid mechanics of the brain. Human brain tissue is a porous, fluid-saturated, nonlinear solid with very small volumetric drained compressibility and negatively charged molecules, capable of permanent deformations. It is a multi-component material with viscous contributions to its solid phase deformation. Several overview articles illustrate the simulation with the above-mentioned model assumptions (Kyriacou et al. 2002), and the development and validation of brain mechanics models (Bayly et al. 2012).

### Biomechanics and mechanobiology

The biomechanical characterization of human brain tissue is a challenging task because of its multiphasic nature, its compliant mechanical response, its multiple modes of loading, and its regional variation of mechanical properties. Another equally important challenge is understanding the brain’s mechanobiology, the reaction of its cells in response to changes in the mechanical environment. In the brain, neuronal signaling is mediated by force-generating proteins. However, the link between endogenous mechanical energy and cellular function has received little attention; yet, a law that summarizes the mechanical consequences of cellular activity would allow us to better understand the relationship between brain structure and brain function (Tyler 2012).

### Literature review

The first dynamical properties of human and Rhesus monkey brain tissues were identified by focusing on the small deformation range (Fallenstein et al. 1969; Galford and McElhaney 1969). In these initial studies, brain parenchyma was viewed as a single-phasic, incompressible, and viscoelastic material. The first large deformation tests on human and Rhesus monkey brain tissues with up to 270 % stretch revealed a nonlinear response with concave upward stress-strain curves (Estes and McElhaney 1970).

Subsequent experimental studies, mainly on porcine and bovine specimens, investigated the large deformation regime in more detail. They observed a strong dependence on the strain rate and a considerable tension-compression asymmetry (Miller and Chinzei 1997, 2002). Further studies revealed a nonlinear viscoelastic behavior (Donnelly and Medige 1997; Hrapko et al. 2006), failure strains under shear deformation (Bilston et al. 2001), shear and compression behavior in the 50–160 % stretch regime (Prange and Margulies 2002), and marked differences between the in vivo and in vitro responses (Gefen and Margulies 2004; Miller et al. 2000). This might—at least in part—be attributed to the considerable amount of residual stress that has been observed in brain tissue in vivo (Xu et al. 2009, 2010).

**Fig. 1:**
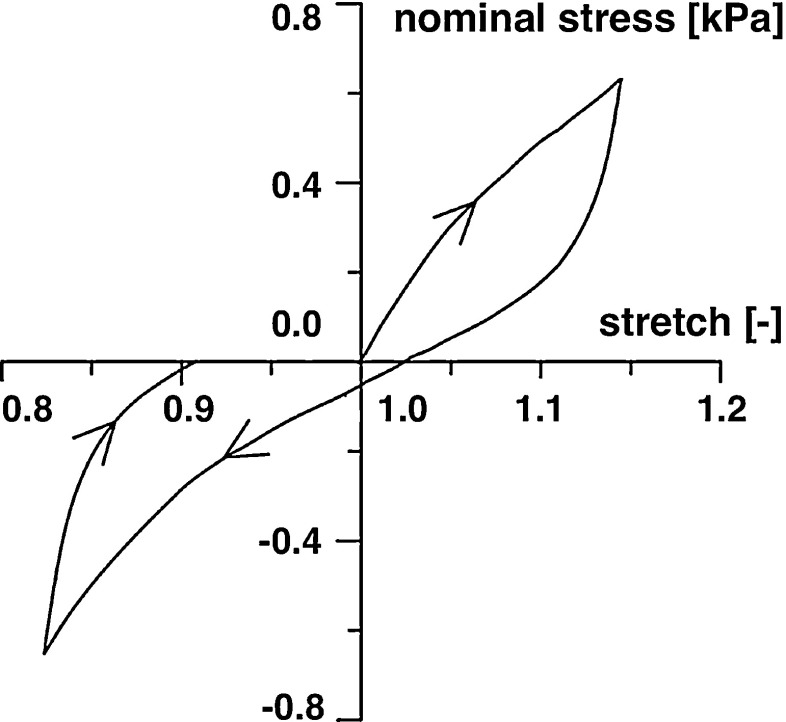
Representative nominal stress versus uniaxial stretch response of tension-compression test below the damage threshold performed on prismatic specimens of human white matter. *Arrows* indicate the loading direction, adapted from (Franceschini et al. 2006)

The systematic study by Franceschini et al. Franceschini et al. (2006) documented in vitro experiments of human brain focusing on white matter tissue. In particular, uniaxial, quasi-static, cyclic tension-compression experiments with a speed of 5 mm/min were performed on 86 cylindrical and prismatic specimens from different orientations and locations within the brain. Figure 1 illustrates the characteristic stress–stretch response of white matter tissue: a peculiar nonlinear mechanical behavior, a typical “S-shaped” curve—similar to materials with filled elastomers—followed by a hysteresis with different stiffnesses in tension and compression and during loading and unloading, and permanent deformations. These typical features of the nominal stress versus uniaxial stretch response were found for all samples. A perfect fit was obtained with a phenomenological model for rubber-like materials, including the Mullins effect and permanent set (Dorfmann and Ogden 2004). When loaded up to failure, softening due to local failure occurred and the shape of the stress-strain curve changed qualitatively (Franceschini et al. 2006).

**Fig. 2:**
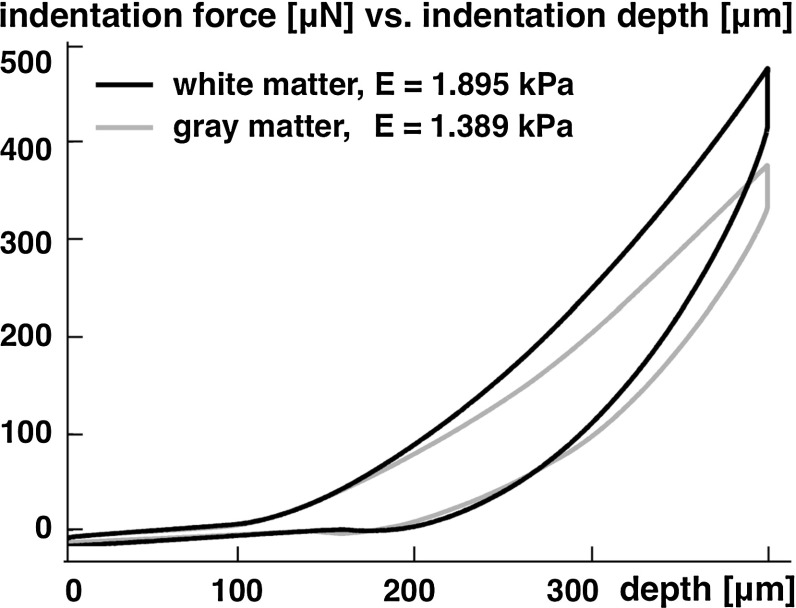
Representative force versus depth response of indentation test performed on coronal slices of bovine white and gray matter. White matter is approximately one-third stiffer than gray matter, adapted from (Budday et al. 2015b)

Another study which focused on aging and regional variations suggested that the adult brain is 3–4 times stiffer than the young brain and that the brain stem is approximately 2–3 times stiffer than gray and white matter tissue (Chatelin et al. 2012). A recent study based on uniaxial tension, compression, and shear tests of human brain samples revealed a pronounced strain rate dependency for all three loading modes (Jin et al. 2013). White matter was stiffer than gray matter in compression and shear, and directional dependency was observed in white matter under shear. Indentation tests of bovine brain tissue revealed that white matter is about one-third stiffer than gray matter (Budday et al. 2015b; Dommelen et al. 2010) as illustrated in Fig. 2. White matter also showed a pronounced anisotropy (Feng et al. 2013; Hrapko et al. 2008; Prange and Margulies 2002; Velardi et al. 2006), displayed larger regional variations than gray matter (Dommelen et al. 2010), appeared to be more viscous, and responded less rapidly to mechanical loading (Budday et al. 2015b).

Recent mechanical characterizations of porcine brain tissue in unconfined compression, simple shear, and tension at dynamic strain rates have motivated constitutive models for brain as a single-phase material to capture these effects (Hrapko et al. 2006; Prevost et al. 2011; Rashid et al. 2012, 2013, 2014).

**Fig. 3:**
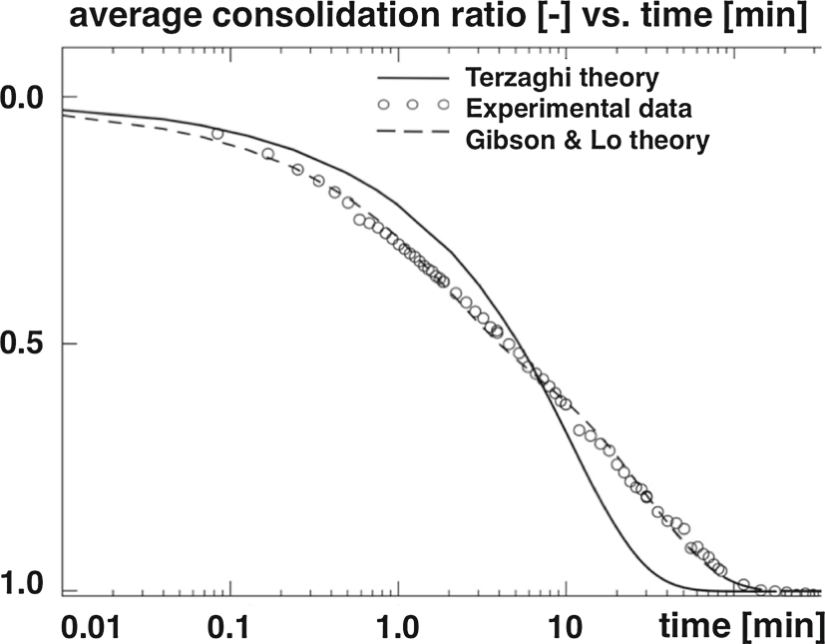
Experimental data obtained from an oedometer test performed on cylindrical specimen of human parietal lobe with free drainage at the top and bottom surface and continuous load steps. The *solid* and *dashed curves* show model results according to the Terzaghi theory and the Gibson and Lo theory, respectively, adapted from (Franceschini et al. 2006)

Alternatively, more advanced approaches consider brain tissue as a poroelastic biphasic material composed of a poroelastic solid phase and a fluid phase, the interstitial fluid. Several physiological instances including volumetric shrinking of brain tissue upon hyperosmotic drug administration point to such a mixed approach (Schrot and Muizelaar 2002) as discussed in Sect. 4. The first data supporting a biphasic theory for brain parenchyma focused on brain tissue mechanics during hydrocephalus (Hakim et al. 1955). Oedometer or consolidometer tests on human brain parenchyma with free drainage at the top and bottom surfaces have shown that brain tissue obeys the consolidation theory with low volumetric compressibility (Franceschini et al. 2006). Experiments under controlled drainage have also revealed the first direct evidence of a poroelastic behavior, similar to fine soils. The temporal evolution of the average consolidation ratio can be modeled using the Terzaghi theory (Terzaghi 1943), a simpler version of the Biot consolidation theory. However, to perfectly capture the consolidation curve in Fig. 3, viscous deformations were included in the model (Franceschini et al. 2006). Confined compression tests on rat brain tissue focused on the influence of the fluid content on the uniaxial compressive deformation. The results suggest that increases in hydrostatic pressure of the extracellular fluid may contribute to brain tissue damage (Haslach et al. 2014). A powerful approach to capture this multi-constituent response can thus be developed on the basis of the theory of porous media (Ehlers and Wagner 2015).

Recent approaches suggest to consider brain tissue as a poroelastic or poroviscoelastic material with negatively charged molecules fixed within cells inducing ion concentration gradients and leading to changes in the intracranial pressure. Triphasic modeling seems reasonable to capture the coupled edematous swelling and mechanical response. In Sect. 4, we will discuss the electrochemistry of brain tissue and its modeling in more detail.

### Open questions and challenges

Despite significant progress, there is a general agreement that a more detailed understanding of brain tissue mechanics is required to develop and improve constitutive models. The mechanical properties of brain tissue, mainly white matter, under specific loading and boundary conditions, have been studied extensively. However, a constitutive model derived under one loading-boundary mode does not necessarily predict the material response under another loading-boundary condition. In addition to unconfined and confined compression, extension, and simple shear tests, supplemental multi-axial tests are required to provide more accurate data toward the material characterization of brain tissue. In particular, triaxial shear tests in addition to biaxial extension tests—performed on the same specimen—would be highly valuable. The design of these experiments should follow the guidelines for independent tests toward reliable tissue characterization (Holzapfel and Ogden 2009). In addition, the investigation of the underlying microstructure of brain tissue is a critical step for future research. This knowledge would allow a more holistic understanding of brain tissue as a single-phasic material. Brain tissue as a biphasic and triphasic material has received less attention in the biomechanics literature. More data are needed on the negatively charged molecules, their charge interactions, and their expression patterns to better characterize phenomena such as brain tissue swelling.

A promising novel approach to characterize the mechanical properties of living brain tissue is magnetic resonance elastography (Kruse et al. 2008; Sack et al. 2008). Magnetic resonance elastography is a noninvasive medical imaging technique to quantify the in vivo shear modulus by applying shear waves to the tissue, imaging the propagating waves, and extracting information using computational algorithms (Hamhaber et al. 2010; Johnson et al. 2013). Restricted to the small deformation regime, with strains smaller than 0.1 % and frequencies larger than 30 Hz, magnetic resonance elastography probes different material characteristics than classical ex vivo tests (Romano et al. 2012). While this technology is still in its infancy, there is hope that it will soon deliver valuable insight into the regional and temporal variation of brain tissue properties during development, aging, and disease progression (Sack et al. 2011). Magnetic resonance elastography has the potential to become a powerful diagnostic tool for various pathologies including multiple sclerosis (Streitberger et al. 2012).

## Fluid mechanics

We now review the fluid mechanics of the brain. The three fluid networks of our brain are the vasculature, the cerebrospinal fluid, and the interstitial fluid. We begin by briefly discussing each of these in turn.

*The Vasculature*. Our brain has very high metabolic demands. A fully functioning cerebral circulation is critical to ensure that blood supply is efficient and that ischemia is avoided. Blood supply to the brain is maintained by a network of collateral vessels and a precise system of autoregulation involving vasodilation and vasoconstriction (Alastruey et al. 2007). The circle of Willis is a ring-like arterial structure located at the base of the brain. The afferent arteries supply blood to the circle, while the efferent arteries transport blood away from the circle. Anastomosing arteries connect the afferent arteries, thus enabling the blood supply to be rerouted to maintain blood flow to the brain, should any afferent blood supply become reduced. Having entered the brain via the circle of Willis, blood then circulates through the microvasculature, where the local exchange of nutrients and oxygen between the blood and surrounding tissue occurs, before leaving through the venous system. To ensure adequate oxygen delivery to the highly metabolically active neurons, the capillary network of the brain is dense. The key distinguishing feature of the brain microvasculature is the presence of tight junctions between the adjacent endothelial cells lining the capillaries. This endothelial layer, referred to as the blood brain barrier, acts as a protective layer separating the blood from the tissue. As the tight junctions prevent transport of substances such as ions and proteins across the blood brain barrier, the endothelial cells actively control the transport of substances crossing the blood brain barrier via ion pumps in their membranes, which enables the composition of the interstitial fluid surrounding the neurons to be well controlled, despite fluctuations in the concentrations of substances in the blood (Abbott et al. 2006).

*The Cerebrospinal Fluid*. The cerebrospinal fluid is a clear plasma-like fluid produced mainly by the choroid plexus. It flows through the ventricular system to the subarachnoid space, where it is absorbed into the blood stream via the sagittal sinus. The cerebrospinal fluid serves many functions, including providing mechanical support for the brain, providing a medium for the transport of humoral messages between regions of the brain, and acting as the waste disposal system for the brain. With these functions, it plays a role similar to the lymphatic system in other tissues (Oreskovic and Klarica 2010). The movement of cerebrospinal fluid through the ventricular system is propelled by the pulsation of the cerebral arteries (Siyahhan et al. 2014).

*The Interstitial Fluid*. The interstitial fluid is an extracellular fluid similar in composition to blood plasma. It fills the interstices of the brain tissue, bathing the neurons. In the healthy brain, only a relatively small amount of fluid is able to leak from the blood brain barrier into the interstitial fluid, due to the low permeability of the blood brain barrier (Abbott et al. 2006). However, if the blood brain barrier is damaged, it can become more permeable to fluid. Additional fluid can cross the blood brain barrier leading to fluid accumulation and swelling in a process known as vasogenic edema. The interstitial fluid undergoes bulk flow, which has several significant implications: non-synaptic cell–cell communication, drug delivery, distribution and clearance, brain ionic homeostasis, immune function of the brain, clearance of $\beta$-amyloid deposits, and cell migration (Abbot 2004).

### Biomechanics and mechanobiology

**Fig. 4:**
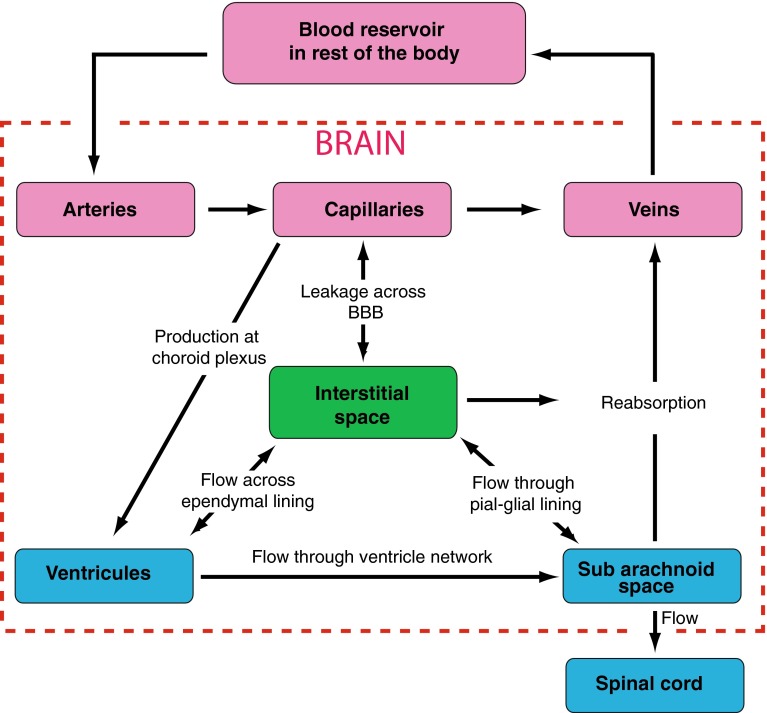
Relationship between the three fluid networks in the brain: the vasculature (*pink*), the cerebrospinal fluid (*blue*), and the interstitial fluid (*green*), adapted with permission from (Lang 2014)

The brain motivates a wealth of exciting problems to study from a physiological fluid mechanics standpoint. Figure 4 illustrates a key feature of intracranial fluid dynamics: the interplay and fluid exchange between the vasculature, cerebrospinal fluid, and interstitial fluid compartments.

One approach to modeling intracranial fluid dynamics is to use lumped-parameter or compartment models, which consider separate compartments for, e.g., the brain parenchyma, vasculature, and ventricles through which fluid is exchanged (Kellie 1824; Ursino and Lodi 1997; Linninger et al. 2009; Jung et al. 2005; Sharan and Popel 2002). While compartment models enable the effect of the system parameters on the fluid flow within the brain to be investigated, they do not allow for consideration of the spatial variation of, e.g., fluid velocity and pressure within an individual compartment. One-dimensional, reduced pulse wave modeling is a good approach accounting for spatial variations when a global assessment of cerebral blood flow is required (Alastruey et al. 2007). This approach can be used to simulate the changes in blood pressure and flow in time and along the axial direction of large vessels. These changes propagate in the form of pulse waves, and carry valuable information about the morphology and functionality of the cerebral vascular system. When detailed two- or three-dimensional spatial information about the nature of the flow is required, an alternative approach is to describe the flow in the vascular and cerebrospinal fluid compartments by the continuity and Navier–Stokes equations for a single-phase Newtonian viscous fluid (Kurtcuoglu et al. 2005; Sweetman et al. 2011; Siyahhan et al. 2014; Kurtcuoglu 2011). These flows can then be coupled to the interstitial flows through the brain parenchyma. The brain parenchyma is a complex composite biological tissue, comprising a wide variety of interacting constituents, including different cell types, their associated extracellular matrix, and interstitial fluid. Multiphase models provide a natural continuum framework to investigate such tissues (Garcia and Smith 2009; Smillie et al. 2005; Levine 1999; Wirth and Sobey 2006; Shahim et al. 2012; Tully and Ventikos 2011; Wilkie et al. 2012; Wirth and Sobey 2009; Stoverud et al. 2012; Linninger et al. 2008; Drapaca et al. 2006). Intracranial flow problems may therefore require the single-phase flow equations for the blood and cerebrospinal fluid domains to be coupled to multiphase flow equations describing interstitial fluid flow in the brain parenchyma via the specification of appropriate boundary conditions between the single-phase and multiphase flow domains. An additional complexity is that the boundaries of the flow domains may be compliant, leading to novel fluid-structure interaction problems where the fluid flows and wall motion are dynamically coupled (Alastruey et al. 2007; Elliott et al. 2013, 2011). Such an approach requires an accurate definition of the vessel wall geometry and the wall structural properties.

What is clear from the above discussion is that modeling flows in the brain leads to exciting physiological flow problems, which can potentially involve the coupling of unsteady, three-dimensional, single-phase and multiphase flows in complex geometries, where the interaction between the flow, the vessel walls, and the tissue leads to novel fluid-structure interaction phenomena. In the following section, we briefly review modeling approaches to understand flows under physiological conditions, as well as those that are encountered as the result of various pathologies, e.g., hydrocephalus and syringomyelia, or clinical interventions, e.g., infusions.

### Literature review

We first summarize models for the vasculature and the cerebrospinal fluid systems in isolation, before introducing global intracranial fluid dynamical models.

*The Vasculature*. A number of studies have focused on the hemodynamics in the circle of Willis (David and Moore 2008; Sforza et al. 2009). A common simplification is to consider rigid vessel walls(Alnaes et al. 2007; Cassot et al. 2000; Cebral et al. 2003; Ferrandez et al. 2001). To determine the influence of vessel geometry on the resulting flow and wall shear stress distributions, computational fluid dynamics methods have been employed to solve the continuity and Navier–Stokes equations where the complex circle of Willis vessel geometry is either idealized (Alnaes et al. 2007; Ferrandez et al. 2001) or imaging techniques are exploited to provide patient-specific geometries (Cebral et al. 2003). An alternative analytical approach is to represent the circle of Willis as an anastomotic network of multiple segments and adopt a simple linear relationship between the mean blood flow in a segment and the pressure difference across a segment to determine the influence of obstructive lesions on the resulting flow (Cassot et al. 2000). A particular challenge when developing circle of Willis flow models is to account for cerebral autoregulation, the vasoconstriction and vasodilation of the small arteries in response to physiological control mechanisms to maintain a relatively constant cerebral blood flow (Paulson et al. 1990; David and Moore 2008). The vessel wall compliance of the circle of Willis has also been modeled exploiting the one-dimensional equations of pressure and flow wave propagation in compliant vessels (Alastruey et al. 2007). Using physiological data, the authors were able to capture the main features of pulse wave propagation along the aorta, at the brachiocephalic bifurcation, and throughout the cerebral arteries.

Cerebral aneurysms are pathological dilations of the arterial walls, driven by a complex interaction of biological and hemodynamic factors. Rupture of cerebral aneurysms can lead to intracranial hemorrhage. The mechanisms of aneurysm formation and evolution, with a focus on the role of hemodynamics, have been extensively discussed (Sforza et al. 2009). It is widely accepted that the wall shear stress exerted by the flowing blood on the endothelial cells lining the arteries plays a pivotal role in the development of aneurysms, motivating computational frameworks coupling the evolution of a cerebral aneurysm to the hemodynamic stimuli acting on the endothelial cells (Watton et al. 2009).

*Cerebrospinal Fluid Flow.* Comprehensive reviews of computational fluid dynamics approaches for the cerebrospinal fluid flow are provided in the literature (Kurtcuoglu 2011). A simplified approach is to consider an idealized geometry of the brain ventricles (Kurtcuoglu et al. 2005). In this initial approach, the domain boundaries were assumed to be rigid, and the flow was driven by the prescribed sinusoidal motion of the third ventricle lateral walls. The model was used to analyze pressure propagation through the system and the influence of a stenosed aqueduct. As an alternative to idealized flow domains, magnetic resonance imaging data have been used to reconstruct patient-specific geometries and physiological boundary conditions (Sweetman et al. 2011). The authors also relaxed the rigid boundary assumption in parts of the domain, considering deformation of the lateral ventricle wall to account for fluid-structure interaction. The model predicted complex cerebrospinal fluid flow patterns and pressures in the ventricular system and subarachnoid space of a normal subject, and the predictions were shown to be in excellent agreement with the subject-specific flow data. Another interesting study used computational fluid dynamics to elucidate the interplay between macroscale and cilia-induced cerebrospinal fluid flows, and their relative impact on near-wall dynamics (Siyahhan et al. 2014). Subject-specific anatomy, wall motion, and choroid plexus pulsations were derived from magnetic resonance imaging data.

*Intracranial Fluid Dynamics*. The above studies focused on the vasculature or cerebrospinal fluid flows in isolation, and provided fundamental insights into the flow conditions in these systems. In many situations, however, it is necessary to consider models of the entire intracranial fluid dynamical system, and we now discuss such models here.

The earliest compartment model for cerebral flow considered three compartments representing arteries, veins, and brain tissue (Kellie 1824). Since this early work, studies have considered an increasing number of compartments, in addition to considering physiological mechanisms such as autoregulation (Jung et al. 2005). The governing equations are derived via conservation of mass and momentum in each compartment, and the fluid fluxes between compartments are driven by hydrostatic and osmotic pressure differences. A recent seven compartment model accounts for arteries, capillaries, veins, brain tissue, cerebrospinal fluid, the sagittal sinus and an artificial compartment for brain swelling and takes into account an autoregulation mechanism, cerebrospinal fluid production, and venous compression (Jung et al. 2005). The model was coupled to a Krogh cylinder model to describe the oxygen supply process. The authors were able to reproduce the experimentally well-established connection between arterial blood pressure and cerebrospinal fluid production. Similar compartment modeling approaches have also been used to consider oxygen transport in the brain microcirculation (Sharan and Popel 2002), and to investigate pathologies such as the onset of edema, hypertension, and hydrocephalus (Linninger et al. 2009).

Alternative approaches, accounting for spatial variations have also been adopted, especially when considering various pathologies, e.g., hydrocephalus and syringomyelia, or clinical interventions, e.g., infusion tests. Such approaches are briefly outlined below.

**Fig. 5:**
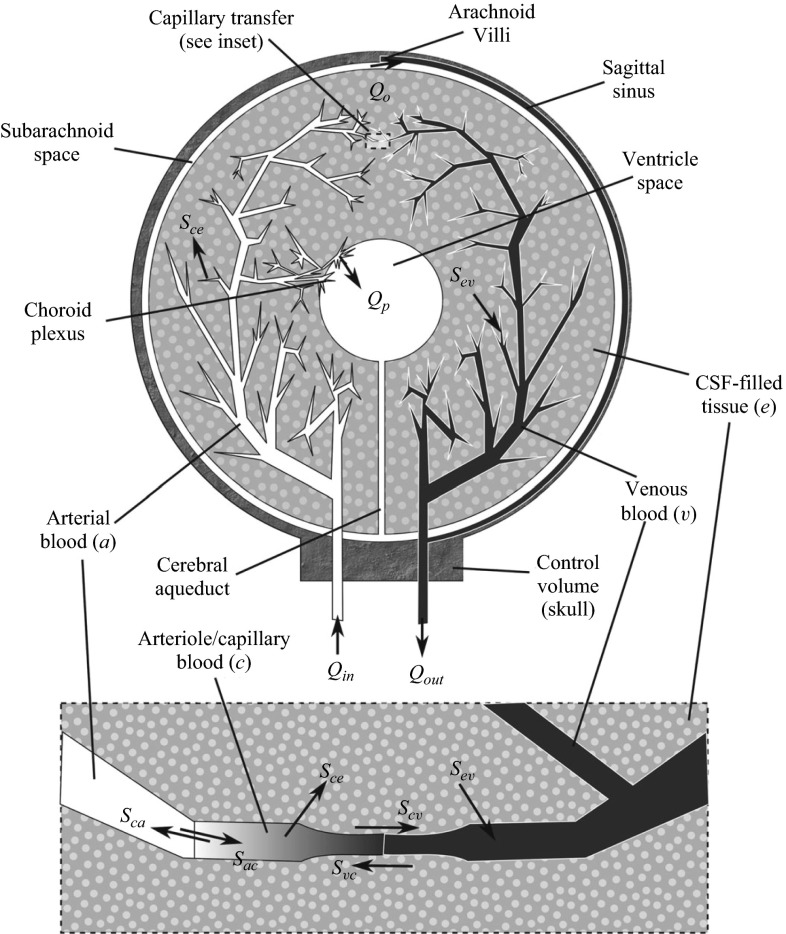
A diagram illustrating cerebral water transport in a multiple-network poroelastic model, reproduced from Tully and Ventikos (2011)

In hydrocephalus, cerebrospinal fluid accumulates in the brain, causing expansion of the ventricles and tissue deformation. The mechanisms leading to hydrocephalus include blockage of the aqueduct of Sylvius, excessive cerebrospinal fluid production, or inhibited cerebrospinal fluid absorption (Clarke and Meyer 2007). Models of hydrocephalus typically couple cerebrospinal fluid flow to the deformation of the surrounding tissue (Wilkie et al. 2012; Drapaca et al. 2006). Poroelastic models for the brain parenchyma, coupled to fluid flow models for the cerebrospinal fluid, have been considered in idealized geometries and time-dependent models characterize the onset, development, and treatment of hydrocephalus (Smillie et al. 2005). The role of absorption of cerebrospinal fluid by the brain parenchyma was also studied (Levine 1999). Both axisymmetric and fully three-dimensional poroelastic models were employed to study the evolution of hydrocephalus (Wirth and Sobey 2006). Healthy and normal pressure hydrocephalus brains were compared in detail (Shahim et al. 2012). Water transport in the cerebral environment was investigated using a multiple-network poroelastic theory in a simplified spherically symmetric geometry (Tully and Ventikos 2011) as shown in Fig. 5. The latter framework allowed detailed investigation of spatiotemporal transport of fluid between the vasculature, cerebrospinal fluid and brain parenchyma, and exploration of hypotheses defining the initiation and progression of acute and chronic hydrocephalus.

A number of studies of hydrocephalus have considered alternatives to poroelasticity. A poroviscoelastic model for brain tissue was adopted to model hydrocephalus and shunting surgery used in its treatment (Mehrabian and Abousleiman 2011). An idealized spherically symmetric brain geometry was used, where the ventricles were assumed to be a hollow concentric space filled with cerebrospinal fluid. A viscoelastic model for the brain parenchyma was used to investigate hydrocephalus, again using an idealized cylindrical geometry (Wilkie et al. 2012). An alternative approach considered a quasi-linear viscoelastic constitutive equation for the brain, again employing an idealized cylindrical geometry, and used the model to determine the decompression and resultant ventricle shrinking due to shunt insertion (Drapaca et al. 2006).

Another pathology of the brain, which involves a fluid-structure interaction problem is syringomyelia (Elliott et al. 2013, 2011). In this condition, one or more fluid-filled cavities, called syrinxes, initiate and develop within the spinal cord. The expansion of the syrinxes causes compression of the surrounding nerve fibers and blood vessels, resulting in neurological damage. Lumped-parameter models of the closed cerebrospinal system were solved numerically to simulate disease conditions and treatment options (Elliott et al. 2011).

Infusion is a procedure during which fluid is injected into the brain, either to administer drugs, which cannot enter from the vasculature as they are unable to cross the blood brain barrier, or to investigate cerebral compliance of the brain. To determine the spatiotemporal distribution of cerebrospinal fluid pressure and brain tissue displacement during an infusion test, a spherically symmetric, three-component poroelastic model of the brain was developed (Wirth and Sobey 2009). A computational fluid dynamics approach was adopted to determine the transport of infused therapeutic agents within the brain (Linninger et al. 2008). Three-dimensional brain anatomy was reconstructed from subject-specific medical images, and tissue anisotropy and heterogeneity quantified using diffusion tensor imaging. Modeling the brain parenchyma as a rigid porous medium, the authors determined the influence of catheter types and positioning on drug dispersion. To determine the concentration of the infused drug and tissue deformation during convection-enhanced drug delivery to brain tissue, patient-specific parameters and geometries from diffusion tensor imaging were combined with a poroelastic model for the brain parenchyma (Stoverud et al. 2012). A biphasic hyperelastic model for the mechanical behavior of brain tissue in a spherically symmetric geometry was used to determine fluid and mass transport, as well as the associated tissue deformation, during convection-enhanced delivery of an infused therapeutic agent (Garcia and Smith 2009).

### Concluding remarks

It is clear that the brain has motivated a wealth of fascinating pathophysiological fluid dynamical problems; yet, a number of challenges remain. While many of the models consider idealized geometries and are able to provide insights into the underlying mechanisms, it is essential to place such models in an anatomically realistic setting, which necessitates an interdisciplinary approach combining the fields of mathematical modeling, scientific computation, and medical imaging. Furthermore, as advances continue to be made in our studying of the underlying physiology and pathology, an additional challenge is to incorporate such biological understanding into the modeling framework, and include, e.g., electrochemical effects within multiphase flow modeling. What is clear is that mathematical modeling of intracranial fluid dynamics is a powerful tool to advance our understanding of the mechanics of the brain.

## Electrochemistry

### Biomechanics and mechanobiology

Electrochemistry plays an important role in many physiological processes and in particular in the mechanics of brain tissues. In most soft tissues, there is a balance between the hydrostatic pressure generated by the microvasculature and the oncotic forces generated by differences in the chemical potentials due to the large molecules such as albumin in the blood plasma. The hydrostatic pressure of the blood vessels is in the range of 1–30 mmHg, whereas the balancing oncotic pressure driving fluid into the circulatory system is in the range of 1–25 mmHg. In comparison, osmotic forces generated by concentration differences of charged particles can be in the range of 1–300 mmHg. In peripheral organs, the small ions are free to move and only a small osmotic pressure is generated by small concentrations of large colloid molecules. The slight imbalance between hydrostatic pressure against osmotic and oncotic pressures drives fluid from the capillary bed into the tissue. This extra interstitial fluid is then removed by the lymphatics.

In the brain, however, the osmotic and hydrostatic balance is completely different. In contrast to most soft tissues, brain tissues are extremely soft. By comparison, a pressure of 30 mmHg corresponds to about 4 kPa which is larger than the typical Young’s modulus for the brain. With a typical leaky capillary bed, as found in the periphery organs, and in the absence of a regular lymphatic system, the fluid pressure would quickly create a very large fluid uptake. This situation, however, does not happen as the capillaries in the brain have tight junctions between endothelial cells called the blood brain barrier, and that is mostly impermeable to ions. Only a small flux of water, of the order of $1 \upmu \text {l}$/min, leaks from the capillaries into the brain tissue and is removed through the ventricles and the subarachnoid space (Redzic et al. 2005). Therefore, overall osmotic effects quantified by osmolality are the dominant forces acting on the fluid and solid phases in our brain. Osmolality is a measure of the concentration of solutes that contribute to osmotic pressure per mass of solvent. It is expressed in Osm/kg, that is, the number of moles of solutes contributing to osmotic pressure per kilogram of solvent. Blood plasma has a typical range of 280–290mOsm/kg, of which only about 1mOsm/kg is due to oncotic solutes. Since the blood brain barrier acts as a semipermeable membrane, the existence of large osmotic gradient will dominate oncotic effects.

*Brain Edema and Tissue Swelling*. The importance of electrochemistry in brain mechanics becomes apparent during brain swelling. Brain edema is the accumulation of water in the interstitium from the capillaries. It is a complex process that can be caused by a number of factors including traumatic brain injury, concussion, ischemic strokes, hemorrhages, infection, tumor growth or even high altitude (Papadopoulos et al. 2004). Brain edemas are usually classified into three categories: interstitial, cytotoxic, and vasogenic (Unterberg et al. 2004). The movement of water in brain tissue depends partly on the hydrostatic and oncotic pressures, the permeability of the capillaries, and the ability of the ventricular network to drain extra fluid; yet, it is primarily regulated by changes in ions and proteins concentration in the tissue. *Interstitial or hydrocephalic edema* results from either the oversecretion of cerebrospinal fluid or its compromised absorption (Unterberg et al. 2004). Therefore, it is mostly the result of an imbalance between inflow and outflow that can lead to acute hydrocephalus, as detailed in Sect. 3. We will therefore focus on ischemia-induced swelling.

**Fig. 6:**
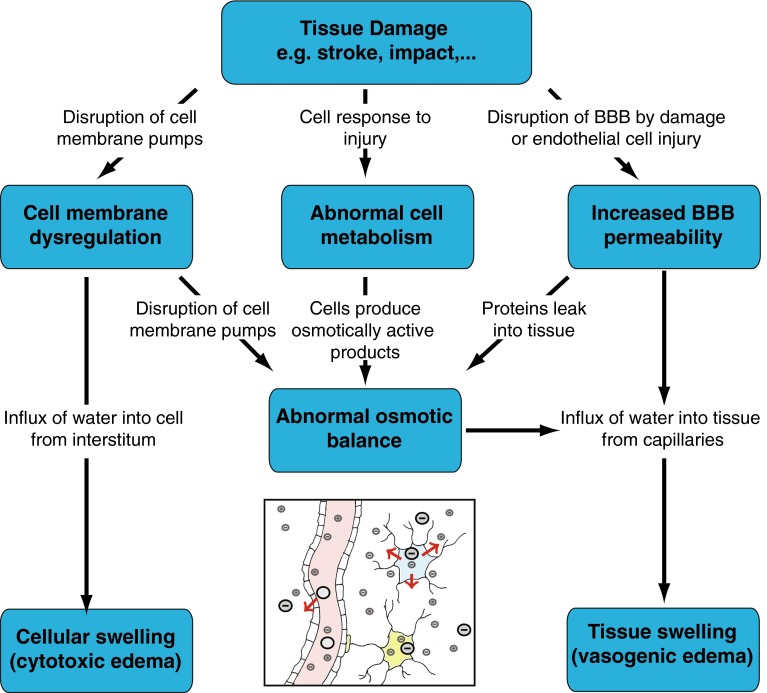
The multiple coupled mechanisms resulting in cytotoxic and vasogenic cerebral edemas. BBB stands for blood brain barrier. The *inset* shows the possible motion of charges in the capillaries, cells, and tissues, adapted with permission from Lang (2014)

During swelling, the fluid enters the tissue from the cerebral capillaries. As shown in Fig. 6, there are several possible mechanisms creating this influx of water. During impact injury, the integrity of the blood vessels can be compromised and blood can directly enter the tissue. After an ischemic stroke, however, it is the ionic imbalance that creates swelling. In a healthy brain tissue, both the composition of the intracellular fluid and the cell volume are actively controlled by ion pumps in the cell membrane. During ischemia, due to a lack of oxygen, the cells cannot properly control the active pumping across the cell membrane, and the osmotic equilibrium is perturbed. As a result, there is a net flux of ions into the cell following electrochemical potential gradients and, accordingly, an uptake of water from the extracellular space into the cell. This cell swelling does not, however, produce tissue swelling as it only trades fluid with the interstitium (Liang et al. 2007). Nevertheless, *cytotoxic swelling* can have a profound effect on the tissue as it changes ion and protein concentrations of the interstitial fluid. This imbalance can provide a driving force for vasogenic edema and lead to bulk swelling (Kawamata et al. 2007). Further, highly swollen cells may burst, causing their contents to become merged with the interstitium. In particular, cells can release negatively charged macromolecules such as glycosaminoglycans and proteoglycans (Syková and Nicholson 2008). Due to their large size, these molecules contribute to the overall *fixed charge density*, that is, the overall negative immobile charges attached to the tissue. The presence of these immobile charges induces an ionic concentration difference between the tissue and the capillary bed, and drives water movement into the tissue through the Donnan effect (Donnan 1924). Under these conditions, the tight endothelial junctions of the blood brain barrier are disrupted and the water influx causes *vasogenic edema*, an overall penetration of fluid in the tissue due to the disruption of the blood brain barrier.

*Treatment and Damage Propagation*. The primary noninvasive treatment for cerebral edema and increase in intracranial pressure is osmotherapy where osmotically active substances such as mannitol or hypertonic salines are administered intravenously to increase serum osmolality (Fink 2012). This change in osmolality creates an osmotic gradient between blood and brain tissue that moves fluids from the interstitium into the intravascular compartment. It is believed that an increase in serum osmolality up to 320 mOsm/l is safe and can reduce brain water content by up to 90 ml (Bhardwaj 2007). In some situations, however, the effect of osmotherapy is limited. For instance, cytotoxic edema that creates an imbalance at the cellular level is resistant to all known treatments (Raslan and Bhardwaj 2007). Similarly, if the integrity of the blood brain barrier is compromised, osmotically active agents will penetrate the tissue and will not be able to create an osmotic gradient. While osmotherapy has an effect in decreasing acute intracranial pressure in many situations, its long-term use and overall benefit is still subject to controversy (Grände and Romner 2012).

**Fig. 7:**
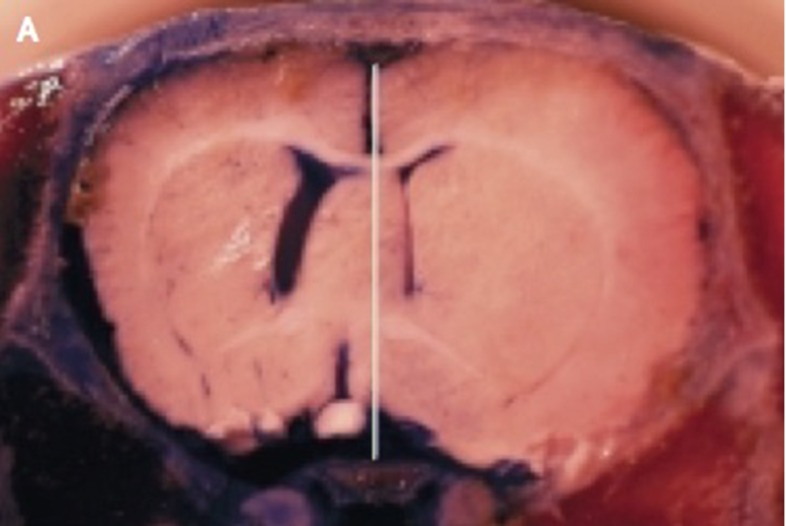
Brain swelling resulting from a middle cerebral artery occlusion. A healthy brain would be symmetric with respect to the *white line*. The midline shift demonstrates extensive swelling of the right hemisphere, reproduced from Simard et al. (2007)

During swelling or increased intracranial pressure, blood vessels can be further impaired due to compression of the surrounding tissue. This load restricts blood flow and, without regulation, increases the ischemic zone and further damages the brain tissue (Walberer et al. 2008). Without treatment, damage can propagate through the brain through the feedback between swelling and ischemia as, for instance, in the middle cerebral artery occlusion shown in Fig. 7. Decompressive craniectomy is used to prevent further swelling and to decrease intracranial pressure. This drastic invasive procedure consists in removing part of the skull to allow the brain to swell (Soustiel et al. 2010). The massive outward swelling creates zones of high stretch which, in turn, can create long-term axonal damage with decreased long-term functional outcome (Cooper et al. 2011). Indeed, axonal stretches as low as 3–5 % have been shown to create internal axonal damage (Chung et al. 2005).

### Literature review

Electrochemical effects at the cellular level are known to play a crucial role in the function and integrity of cells, and kinetic models are used to determine cellular ionic balance. Cytotoxic edema resulting from strokes has been investigated by combining kinetic models with compartment models for the fluid components (Dronne et al. 2006). In these models, both passive and active phenomena, such as cell membrane pumps, are taken into account to compute the flux of ions. The resulting osmotic pressure gradients drive the fluid between the intracellular and extracellular fluid compartments and explain the mechanism of cell swelling.

The coupling between electrochemical effects, fluid transport, and elastic deformation is particularly difficult and is only little understood, specifically in the brain. A possible modeling approach to take into account these coupled effects is to use the theory of mixtures. The fundamental idea from mixture theory, based on the early work of Truesdell and Bowen Bowen (1967), is that each phase has its own identity and velocity but both phases coexist at any spatial point and contribute to the overall physical properties of the system such as density and stress. However, it is the overall macroscopic quantities that must satisfy the physical laws such as the conservation of mass or the balance of stress given by Cauchy equation so that individual phases can exchange density and linear momentum in a pointwise fashion.

Electrochemical effects are particularly important for the modeling of articular cartilage, for which there is a large body of literature (Lai et al. 1991; Huyghe and Janssen 1997; Ateshian et al. 2006). In this approach, a tissue is modeled as a mixture of three or more phases: a solid phase representing the elastic contribution of the extracellular matrix and cells, a fluid phase representing the interstitial fluid, and phases to include the solute and ion species in solution. Fixed charge densities can be easily modeled by assigning a net charge to the solid phase and the thermodynamic balance coupled to the mechanical balance in these systems naturally explains osmotic behaviors such as the Donnan effect. One of the main issues in these *triphasic* or *quadriphasic models* is to provide reasonable constitutive laws for the drag between phases. For instance, the solid-fluid interaction can be modeled by Darcy’s law and the fluid-solute interaction can be modeled by Fick’s law. The drag between solid and solute phases is more subtle and a law must be chosen to model effects related to reduced diffusion of large solutes passing through a soft solid phase as observed in brain tissue (Nicholson 2001).

**Fig. 8:**
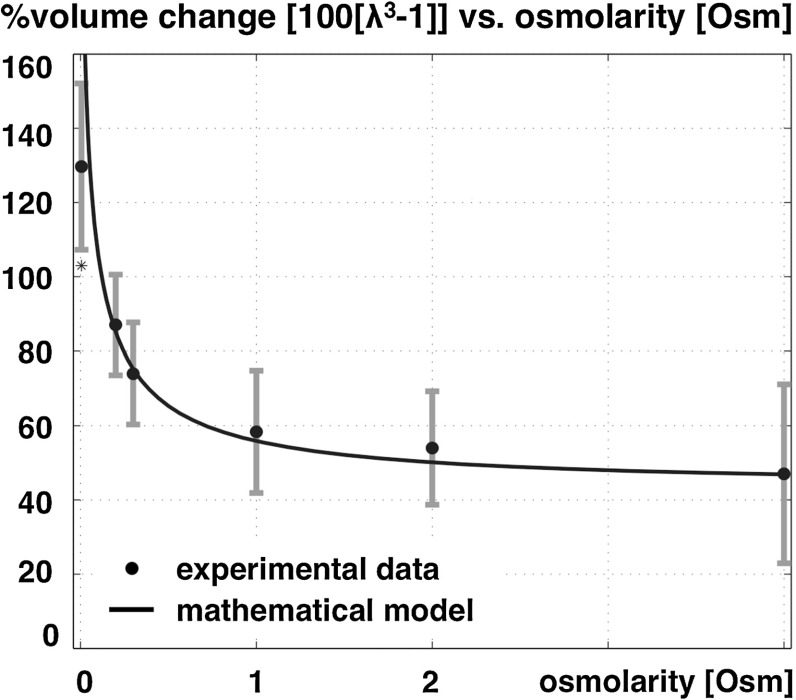
Experimental swelling data of tissue slice as a function of bath ionic concentration [data from (Elkin et al. 2010)] and curve fit of the volume change of dead rat brain tissue as a function of bath osmolarity. The *error bars* allude to the minimum and maximum range based upon the standard error of the mean, adapted from Lang et al. (2014)

To study brain tissue and demonstrate the importance of fixed charge density during damage, the first quadriphasic model combined theory and experiments of healthy and damage brain slices in different solute concentrations (Elkin et al. 2010). Figure 8 shows experimental data of the free-swelling volume change of dead rat brain tissue as a function of bath ionic osmolarity.

These experimental results were reinterpreted within a quadriphasic theory, suggesting that the observed swelling cannot be explained by the Donnan effect itself as it would imply unrealistically low tissue stiffness or unrealistically high fixed charge density (Lang et al. 2014). However, the observed swelling can be fully explained by taking into account the presence of non-permeating solutes. Non-permeating solutes are large uncharged solutes, which cannot travel through the mixture due to mechanical and geometric restrictions, but contribute to the overall osmotic pressure difference. The solid curve in Fig. 8 indicates the best fit model using a quadriphasic mixture theory including non-permeating solutes for fixed charge density and an elastic material parameter independently fitted. This enhancement provides a satisfying agreement with experimental data (Elkin et al. 2010). A quadriphasic theory was also used to model hydrocephalus by driving tissue swelling through an ionic change in the cerebrospinal fluid (Drapaca and Fritz 2012).

Surprisingly, there has been little work on damage propagation and deformation of the brain caused by craniectomy. A first theoretical model in spherical geometry couples the diffusion field with cell death, release of fixed charge density, and swelling (Lang et al. 2015). The model revealed that for a large enough initial damage zone, this feedback causes damage to propagate through the entire brain in a closed skull. However, damage can be halted by decompressive surgery at the expense of axonal stretching. Biomechanical issues related to tissue deformation during decompressive craniectomy have also been addressed experimentally and computationally by finite element modeling (Gao and Ang 2009; Fletcher et al. 2014).

### Open questions and challenges

Brain deformation and stress are tightly regulated by fluid, solid, and electrochemical effects. Therefore, mathematical models of brain tissue swelling must include these different components to address a number of key phenomena such as the nonlinear elastic response of brain tissue, its porous behavior, water accumulation, Donnan effects, hydrostatic and osmotic pressure-driven deformation, deformation-dependent permeability, and diffusion restricted by the solid phase. In principle, quadriphasic theories are able to include all these effects and therefore provide an accurate model of the macroscopic mechanical environment in the brain. However, these models are inherently complex, built on shaky fundamental principles, and their constitutive laws are not yet properly validated or justified from first principles. At a theoretical level, the development of homogenization methods for coupled fields in large deformations may offer a systematic way to justify these models. Independently, quadriphasic models offer a general modeling framework suitable for a combined phenomenological and computational approach. These models need to be combined with realistic geometries and realistic fiber orientations, e.g., from diffusion tensor magnetic resonance imaging.

Quadriphasic models only describe the macroscopic behavior of the tissue—they must somehow be related to the microscopic behavior at the molecular or cellular levels. Therefore, a multi-scale approach will be necessary to bottom-up inform macroscopic phenomenological models from the microstructure, but also to top-down extract information from the continuum level to predict behavior at the neuron level such as axonal damage. The combination of these methods could create a unique platform to address key scientific and clinical issues related to brain swelling, trauma, and surgery.

### Concluding remarks

A number of questions related to the mechanics in the healthy brain can be addressed from a plain solid or fluid perspective as discussed in Sects. 2 and 3. However, when trauma occurs due to brain injury, strokes, or other processes, charged molecules and ions are in imbalance and cannot be neglected. In these circumstances, electrochemical effects play a crucial role in the overall balance of stresses, the intake of fluids, and tissue deformation. Moving forward, an understanding of brain mechanics must combine these multiple phases and must be informed by subcellular and cellular phenomena at the neuron level.

## Electromechanics

### Biomechanics and mechanobiology

Neurons, along with glial cells, e.g., astrocytes and oligodendrocytes are the major cell types in the brain. The Greek word glia, $\gamma \uplambda \iota \alpha$, literally means glue. Glial cells do not conduct electrical signals, and their role in the brain was originally thought to be restricted to structural support and mechanical protection for neurons. However, they are now known to participate actively in the growth, migration, regulation, and healing of neurons through various mechanisms including synaptic connection regulation and protein synthesis (Pfrieger 2010). This initial misconception highlights the need to study conjointly electrophysiology, biophysics, and mechanics when tackling problems such as brain development, damage, and healing.

### Literature review

Many current research activities aim at understanding brain function from the protein to the functional scales with the goal to establish direct relationships with well-known diseases. However, today’s major research campaigns focus primarily on the electrophysiological nature of this problem and almost entirely neglect the role of mechanical effects. Yet, in parallel to these efforts, recent findings suggest that major brain development mechanisms and diseases are directly linked to the mechanical response of the brain. Traumatic brain injury, spinal cord injury, Alzheimer’s disease, and cancer have all shown to be dependent on the mechanical behavior of neurons and glial cells (Suresh 2007; Ouyang et al. 2010; Goldstein et al. 2012).

The long time scale involved in the evolution of dementia and cancer implies that the coupling between biochemical and mechanical properties of cells is extremely complex to establish either experimentally or numerically. Nevertheless, it has now been establish that traumatic brain injuries (and especially repeated injuries) play a role in the long-term evolution of diseases through post-traumatic epilepsy (Kharatishvili et al. 2009) or tauopathies (Goldstein et al. 2012). Short-term events such as traumatic brain injury and spinal cord injury, however, provide quasi-immediate alterations of both mechanical and biochemical properties of the injured cells. Additionally, after a long tradition of research focused exclusively on mechanical criteria, mechanical injuries are now the subject of new research efforts, both experimentally and numerically, with the goal to evaluate the resulting electrophysiological alterations. In this section, we present these advances by focusing primarily on traumatic brain injury and spinal cord injury and their effects on individual neurons.

*From mechanical insult to electrophysiological deficit.* During the propagation of a pressure wave in the cranium cavity, e.g., from blast or head impact or the sudden stretch or compression of the spinal cord, neuronal cells are subjected to either an intense overpressure that occurs within microseconds or a large strain followed by immediate relaxation. Studies of cellular response after blast and strain events provide evidence of degenerative processes in the neuron itself (Leung et al. 2008; Duncan et al. 2011). However, most of the experimental efforts to study this phenomenon at the cellular level generally overlook its chemical and electrical properties crucial to fulfill its basic mission. Again, the neuronal electrical properties are inherently relying on the structural cell components—in this case, the membrane’s ability to exchange ions with the extracellular matrix (Koch 1999). The functional evaluation of cell injury as caused by strain or pressure gradients, either temporal or spatial, thus intrinsically depends on the membrane and the cytoskeleton’s mechanical cohesion.

*Experimental efforts.* Neuropathological and neurological traumatic brain injuries and spinal cord injuries ranging from simple edema or axonal injury to cognitive deficits or post-traumatic stress disorders have been widely studied in both animal models and humans (Hicks et al. 2010; Duncan et al. 2011; Goldstein et al. 2012). At the cellular level, these injuries have been more specifically linked to cytoskeleton alterations, neurotransmitter deficits, disruption of calcium homeostasis, increase in synaptic calcium influx, mitochondrial disturbances, loss in membrane permeability, blood brain barrier dysfunction, demyelination, axonal swelling, diffuse axonal injury, and even altered gene expression (Smith et al. 2003; Cernak and Noble-Haeusslein 2010; Ouyang et al. 2010; Alford et al. 2011; Peter and Mofrad 2012; Spaethling and Meaney 2012; Hue et al. 2013).

Some purely mechanistic damage criteria have been proposed (Smith et al. 1999), but it is only recently that a link has been made between mechanical properties and function. A first study evaluated visual evoked potentials in guinea pig optic nerves before and after stretch (Bain and Meaney 2000). In guinea pig spinal cord white matter, the injury level was quantified by measuring membrane integrity and compound action potential propagation under ex vivo electrical stimuli after tensile (Shi and Whitebone 2006), compressive (Ouyang et al. 2008) and blast loading (Connell et al. 2011). At the protein scale, leaky voltage-gated sodium ion channels were observed after trauma. The degree of trauma of these Nav1.6 ion channels was quantified by the shift in the conditioning potential necessary to recover the pre-trauma current characteristics (Wang et al. 2009). However, this left-shift potential was not directly related to mechanical parameters, but rather based on a direct modification of electrophysiological properties.

*Numerical efforts.* Neuronal electrochemical interaction properties, especially at the membrane level, are relatively well described thanks to the pioneering work of Hodgkin and Huxley Hodgkin and Huxley (1952). This model and its derivatives involve the consideration of currents and ion concentrations through and along the neuronal membrane (Koch 1999; Shepherd 2004). Computational simulations making use of finite element schemes allow us to identify stress extrema and stress profiles during traumatic brain injury at the cellular level (Jérusalem and Dao 2012) and at the tissue level (Moore et al. 2009; Nyein et al. 2010; Cloots et al. 2013; Gupta and Przekwas 2013; Jean et al. 2014). However, these efforts generally only provide a set of mechanical criteria for the observed damage, and a direct link between mechanical quantities and functional alterations is missing.

To this end, a model for the hyperpolarization/left shifts of the ion channel current was proposed (Boucher et al. 2012) to capture the experimentally observed effects (Wang et al. 2009). This model successfully reproduces the effect of trauma-induced blebbing on the electrophysiological properties of a membrane patch. Building on the same left-shift mechanism, a similar study indicated a marked difference in the behavior of potentials in subthreshold, $<$14 mV of potential shift, and supra-threshold, $>$14 mV, traumas (Volman and Ng 2013). Other approaches focusing on demyelination, a classical hallmark of multiple sclerosis, and its geometric effect on the additional exposition of the membrane to the surrounding medium date back to the 1970s (Waxman and Brill 1978) and have been extended to explore the effect of drug treatment on the conduction in the damaged region (Babbs and Shi 2013). Despite a growing interest in linking trauma and electrophysiological alterations, the intrinsic relation between mechanogeometrical phenomena including stress, strain, and strain rates, and electrophysiological alterations including potentials and currents has not been included in these models.

Recently, a model that directly links macroscopic strain and strain rate to functional deficits in guinea pig white matter spinal cord during stretch loading was proposed (Jérusalem et al. 2014; García-Grajales et al. 2014). This model consists of three components: a mechanical model to scale down any macroscopic strain to the microscopic membrane level, an electrophysiological model to describe the electrical propagation within the axon, and a coupling model to link the microscopic strain by defining two types of alterations: geometric, i.e., due to geometric changes during stretch, and damaging, i.e., due to an excess of strain at the membrane level leading to membrane and ion channels failure.

### Concluding remarks

While it is now appreciated that mechanics can dramatically alter the electrophysiological response of neurons in the short and long terms, the simultaneous measurement of electrophysiological and mechanical properties of the mammalian brain remains an extremely challenging problem. However, mathematical and computational models can be used as support tools to characterize the electromechanical features of the brain across multiple scales of observation to gain a global insight into brain function in health and disease. This will eventually allow us to establish a link between cellular damage at the microscale, functions at the mesoscale, and behavioral alteration at the macroscale.

## Brain development

### Biomechanics and mechanobiology

For a long time, the development of the mammalian brain was viewed as a purely morphogenetic process, independent of forces, stress, stretch, or strain. There is now increasing evidence that mechanical factors such as thickness, stiffness, and growth play a significant role in regulating gyrification, the process of brain folding. A better understanding of the mechanisms that drive brain folding may have direct implications on the diagnostics—and possibly treatment - of neurological disorders such as schizophrenia or autism.

### Literature review

For more than a century, the unique surface morphology of the mammalian brain has fascinated scientists of all disciplines alike (Welker 1990). Recent developments in medical imaging reveal that the folding pattern of our brain is not only associated with intelligence, but is also closely correlated with neurological dysfunction (Raybaud and Widjaja 2011).

**Fig. 9:**
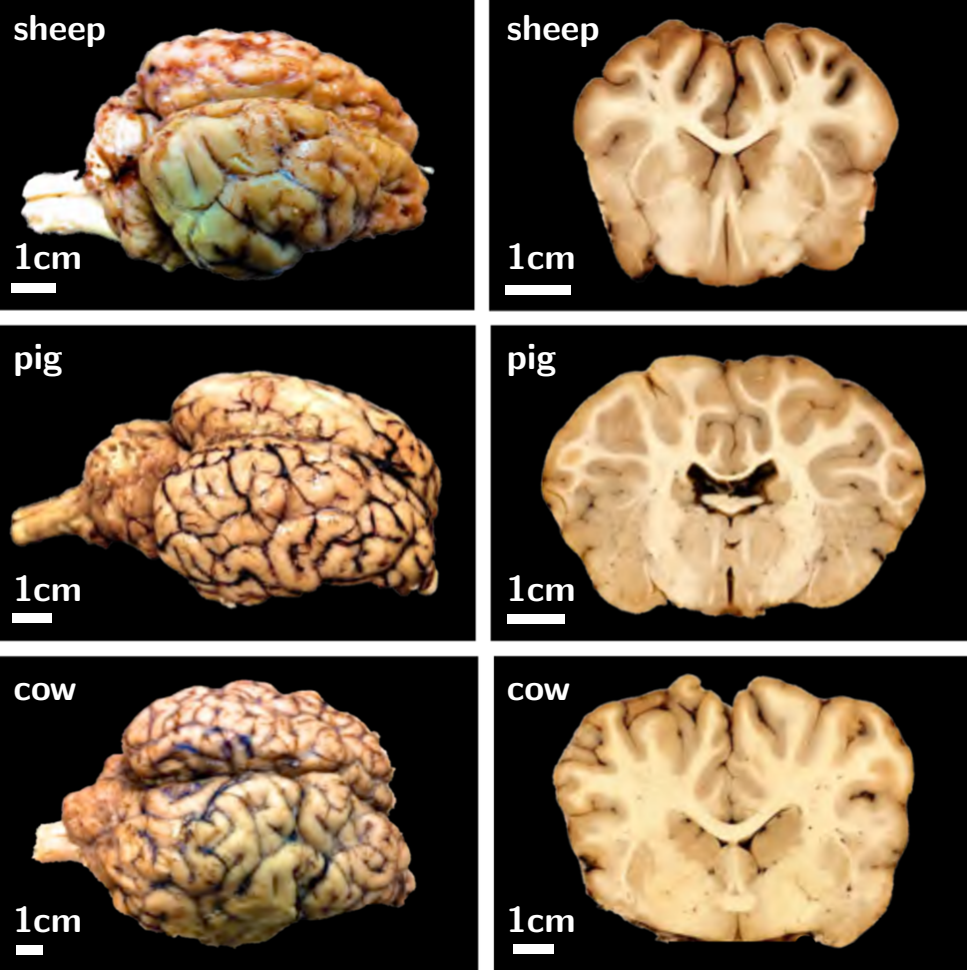
Surface morphology of the mammalian brain. Larger mammals have larger and more folded brains: The bovine brain (*bottom*) is larger and more folded than the porcine brain (*middle*) which is larger and more folded than the ovine brain (*top*). The cortical thickness is relatively similar in all mammals

Figure 9 illustrates the characteristic surface morphology of the mammalian brain. The photographs of bovine, porcine, and ovine brains reveal two important characteristics: Larger mammals tend to have larger brains and larger brains tend to be more folded than smaller brains (Zilles et al. 2013). With a volume of 1200 cm$^3$, a surface area of 1800 cm$^2$, and a cortical thickness of thicknesses of 2.5 mm, the human brain is one of the largest and most folded brains. The ratio between brain surface area and volume, and with it the degree of gyrification, varies significantly between species (Welker 1990). Yet, as the frontal coronal sections in Fig. 9 indicate, the thickness of the outer layer remains remarkably well preserved: It varies by less than an order of magnitude across all species.

The mammalian brain possesses an outer cortical layer of gray matter, which contains primarily cell bodies, and an inner subcortical core of white matter, which contains the axons that connect them. The development of this distinct microstructural architecture takes place in two stages, which are crucial for cortical folding (Roth and Dicke 2005): First, progenitor cells located around the ventricles divide symmetrically into two new progenitor cells to increase the total number of cells in the brain; second, these newly created cells divide asymmetrically into a progenitor cell and a neuron (Sun and Hevner 2014). All neurons of the same progenitor cell migrate outwards and form a cortical column (Hatten 1999). Accordingly, symmetric division is closely related to a growth in surface area, while asymmetric division is closely related to a growth in cortical thickness (Roth and Dicke 2005). During later stages of development, tangential expansion is associated with the maturation of the neocortex caused by an increase of neurons in size, the formation of cortico-cortical connections, and the addition of intracortical glial cells (Welker 1990).

Pathological cell division or cell migration can cause abnormalities in surface area or thickness (Hatten 1999). Prominent examples are polymicrogyria, associated with a large number of short and small folds that can increase surface area, and lissencephaly, associated with an increased cortical thickness and a small number of long and shallow folds (Raybaud and Widjaja 2011). Severe malformations are often correlated with developmental delay, epilepsy, schizophrenia, and autism.

The first mechanical model that explains brain development dates back almost four decades (Richman et al. 1975). It attributes cortical folding to differential growth, a mechanism to release growth-induced residual stresses by surface buckling. While the model predictions agree well with stress distributions from dissection experiments, they rely on an unrealistically large stiffness ratio between the cortical and subcortical layers (Bayly et al. 2014). To address these shortcomings, an alternative model was proposed more than two decades later (Essen 1997). It attributes cortical folding to axonal tension, a mechanism that brings functionally related units topographically closer together. While this second model explains folding irrespective of the stiffness ratio, it disagrees with the stress distributions from dissection experiments (Xu et al. 2010). The controversies around these two models have raised the question whether mechanics is suitable at all to explain the mechanisms of cortical folding.

Recent progress in extreme mechanics of growing matter holds promise to provide new insight into the developing brain. An emerging trend is to combine both approaches to account for differential growth between cortex and subcortex in combination with chronic axon elongation in the subcortex (Bayly et al. 2013). Using the nonlinear field theories of mechanics supplemented by the theory of finite growth, the latest brain models characterize the developing brain as a morphogenetically growing outer layer on a mechanically growing inner core (Budday et al. 2014).

To establish analytical estimates for the critical folding pressure and the critical wavelength, we can interpret cortical folding as the instability problem of a confined, layered medium subjected to growth-induced compression (Biot 1957). The classical Föppl–von Kármán theory then allows us to characterize cortical deflection through a fourth order plate equation (Bayly et al. 2013). With a sinusoidal ansatz for the cortical deflection and a Maxwell-type viscoelastic ansatz for the subcortical transverse force, we can relate the gyral wavelength to the cortical thickness, stiffness, and growth rate (Budday et al. 2015a). Since the absolute cortical stiffness and growth rate are poorly characterized, it has become common practice to explore the role of the relative cortical stiffness and growth rate with respect to the subcortical properties.

**Fig. 10:**
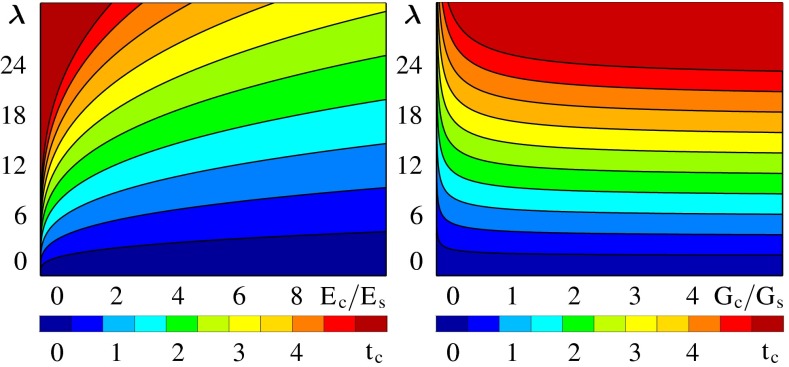
Analytical estimates of the brain surface morphology. The gyral wavelength $\uplambda$ increases with increasing cortical thickness $t_\mathrm{c}$ (from *blue *to *red*), increasing stiffness ratio between cortex and subcortex $E_\mathrm{c}/E_\mathrm{s}$ (*left*) and decreasing growth ratio between cortex and subcortex $G_\mathrm{c}/G_\mathrm{s}$ (*right*)

Figure 10 shows analytical estimates for the brain surface morphology for varying cortical thicknesses, and varying stiffness and growth ratios between cortex and subcortex (Budday et al. 2014). The graphs reveal that the gyral wavelength, the distance between two neighboring gyri, is directly proportional to the cortical thickness, to the third root of the stiffness ratio between cortex and subcortex, and to the subcortical growth rate. For the two extreme cases of slow and fast subcortical growth, the subcortex behaves either solid- or fluid-like. While a solid-like subcortex has no affect on the gyral wavelength, a fluid-like subcortex can significantly increase the gyral wavelength.

To explore the progression of gyrification beyond the onset of folding, we can adopt the continuum theory of finite growth (Ambrosi et al. 2011). This theory consists of a set of five equations, which define the kinematics, the constitutive behavior, the mechanical equilibrium, the growth kinematics, and the growth kinetics (Menzel and Kuhl 2012). Kinematically, the key ingredient is the multiplicative decomposition of the deformation gradient into an elastic and a growth part. Constitutively, only the elastic part of the deformation gradient enters the formulation of the hyperelastic free energy, which we introduce to define the stresses. Dynamically, these stresses enter the mechanical equilibrium equations. In addition to these standard continuum equations, we need to define the kinematics and kinetics of growth (Papastavrou et al. 2013). Kinematically, it is common to assume that growth is either isotropic or anisotropic with a preferred direction. In either case, we can parameterize growth in terms of a single, scalar-valued growth multiplier. Kinetically, it seems reasonable to model cortical growth as purely morphogenetic and subcortical growth as either stress driven (Bayly et al. 2013) or strain driven (Budday et al. 2014).

**Fig. 11:**
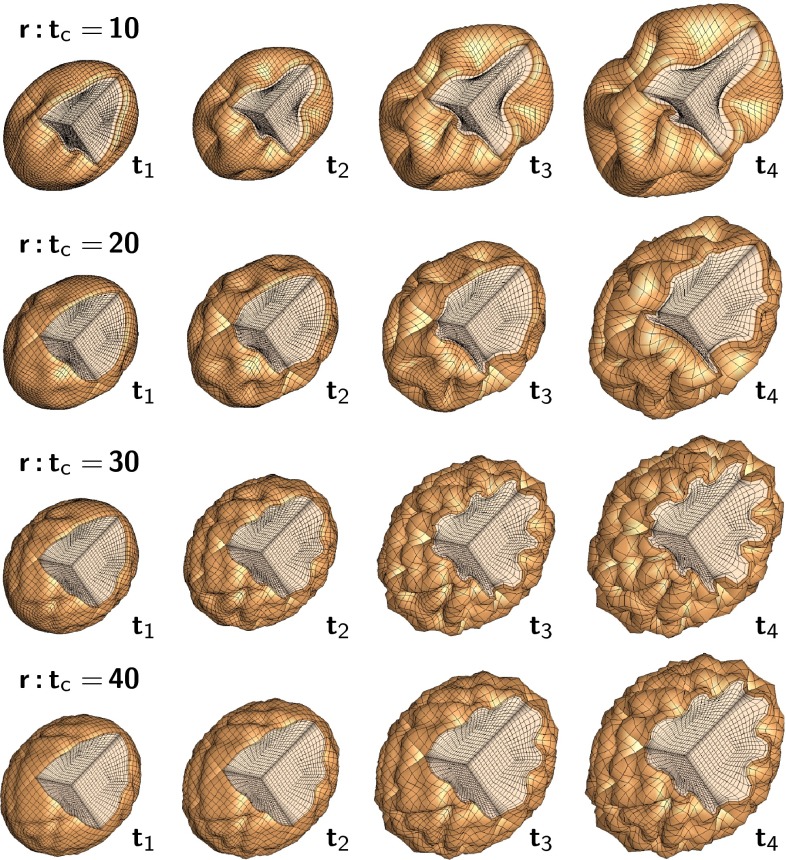
Computational modeling of brain surface morphology. Keeping the thickness of the cortical layer constant, we observe that larger brains tend to be more folded. Indeed, as the radius-to-thickness ratio increases from $r:t_\mathrm{c}=10$, top, to $r:t_\mathrm{c}=40$, bottom, the brain surface becomes more convoluted as time evolves from $t_1$ to $t_4$

Figure 11 illustrates a computational simulation of gyrification for varying radius-to-thickness ratios. The idealized ellipsoidal brain model predicts the gradual formation of gyri and sulci. Folding is initiated first along the long axis of the ellipsoid where the curvature of the ellipsoid is lowest (Budday et al. 2015c). As time evolves, folding gradually propagates outward. Once the folding pattern has formed, gyri and sulci grow deeper until they begin to form contact. Computational modeling not only confirms our analytical estimates, but also predicts the formation of complex surface morphologies with asymmetric patterns and secondary folds. The simulation explains the observation that larger brains are more folded than smaller brains: As the brain size increases, the surface becomes more convoluted. The model also explains why lissencephalic brains with an increased cortical thickness have a smooth surface with a small number of large folds and why polymicrogyric brains with an increased surface area have a large number of small folds. Thickening of the leptomeninges overlying the malformed cortex is seen in 80 % of cases. This may impose physical constraints on the developing cortex, forcing it to undergo increased folding in order to be accommodated in an area where expansion is limited by the rigidity of the overlying leptomeninges (Squier and Jansen 2014).

### Open questions and challenges

Recent developments have shown that mechanical modeling can explain cortical folding during brain development (Budday et al. 2014). Both analytical and computational models support the hypothesis that mechanical features such as the cortical thickness, stiffness, and growth are important regulators of gyrification (Bayly et al. 2013). This relationship is clinically important since modern imaging techniques reveal a close correlation between brain form and function (Raybaud and Widjaja 2011). In severe neurological disorders such as schizophrenia and autism, specific regions of the brain display significant microstructural alterations correlated with pathological morphologies. Remarkably, either pharmacological treatment or behavior therapy can alter—and possibly revert—these malformations over time. Mechanical modeling could serve as a valuable tool to systematically characterize healthy brain surface morphologies, identify pathological alterations, and, ultimately, guide the design of treatment strategies for neurological disorders.

Analytical modeling provides a quick first insight into the critical conditions at the onset of folding; yet, it fails to predict the evolution of complex instability patterns in the post-critical regime (Ben Amar and Goriely 2005). Computational modeling allows us to predict realistic three-dimensional surface morphologies at the onset of folding and beyond (Menzel and Kuhl 2012). However, the calibration and validation of analytical and computational models remain a major challenge. In humans, cortical folding takes place in utero during 24 and 32 weeks of gestation. In preterm infants, cortical folding is incomplete at birth, which allows us to explore the evolution of folding patterns using magnetic resonance imaging (Raybaud et al. 2013). Recent trends in clinical imaging indicate that we may soon be able to image cortical folding noninvasively in utero. Mechanics could play a central role in translating these images into quantitative diagnostic predictors of brain function.

To increase confidence in mechanical modeling, in addition to a thorough, systematic mechanical characterization (Budday et al. 2015b), we would need longitudinal sequences given by multiple images of the same brain in time. In the ferret, where folding takes place postnatally, longitudinal imaging studies have been performed to inform model design and model calibration (Knutsen et al. 2010, 2013). In humans, where folding is almost completed upon birth, longitudinal information is mainly based on brain atlases, merged images from a large number of scans of preterm infants (Raybaud et al. 2013). While those averaged images provide excellent insight into the common overall features of the human brain, they smooth out local perturbations such as secondary and tertiary folds, which are typical indicators of some neurological disorders (Raybaud and Widjaja 2011). Multiple scans of a single individual at different time points throughout the gyrification process would be critical to interpret local variations in cortical folding and identify mechanisms of malformation.

Computational modeling is an ideal tool—if not the only one—to bridge the scales and correlate subcellular and cellular events such as axonal migration and axonal elongation with clinically relevant characteristics such as cortical thickness, surface area, and gyrification indices. Yet, most existing growth models are still purely phenomenological as they characterize growth through a single growth multiplier without a clear microstructural interpretation (Menzel and Kuhl 2012). By incorporating the underlying biology and biochemistry, plain mechanical models could be made more mechanistic (Budday et al. 2014). For gray matter, we could correlate surface growth to symmetric progenitor cell division and thickness growth to asymmetric cell division (Hatten 1999). This effect suggests a modification in the kinematics and kinetics of growth: First, the growth tensor should not be considered as isotropic, but rather as transversely isotropic with independent surface and thickness contributions; second, these two mechanisms could be modeled independently in time and be driven by different biochemical cues. For white matter, we could correlate growth to chronic axon elongation (Bray 1984). This would suggest a regionally varying anisotropic growth tensor to allow for growth along the principal axon orientation and a growth multiplier that could be tied to chronic axon elongation experiments (Holland et al. 2014).

### Concluding remarks

Mechanical factors are increasingly recognized as important regulators of brain morphology. Recent studies indicate that analytical and computational modeling can explain physiological gyrogenesis and pathological malformations. Understanding the process of cortical folding in the mammalian brain has direct implications on the diagnostics—and possibly treatment—of neurological disorders including severe retardation, epilepsy, schizophrenia, and autism.

## Brain tumors

The past two decades have witnessed a significant growth in research on how the mechanical and biophysical properties of cells and subcellular structures influence the growth and progression of cancers. In recent years, the application of continuum mechanics to biology and the biomedical sciences has provided a broad framework that has led to invaluable insights and advances in many areas of the biomedical sciences. As a result, researchers have started to apply these methods and approaches to study the biomechanics and mechanobiology of cancer (Suresh 2007).

Cancer is a disease that arises from dysfunction of cells, resulting in uncontrolled proliferation, leading to disruption of tissue and organ function. In particular, brain tumors are atypical, aberrant aggregates of abnormal cells that form in the brain parenchyma of the central nervous system. The most aggressive of the tumors, clinically classified as gliomas, is the Grade IV glioblastoma multiforme. Glioblastoma multiforme appear to have a peak occurrence in the 45–60 year old demographic and present radiologically as a grossly heterogeneous mass, with ring enhancement around a necrotic core. This zone is often surrounded by vasogenic edema, and accompanied by signs of hemorrhage. The multi-scale heterogeneity—from the molecular via cellular to tissue-level manifestation of glioblastoma multiforme—may lie at the heart of why this type of cancer is so resistant to therapeutic interventions. Almost a century ago, radical interventions such as surgical hemispherectomy proved ineffective. Even after a century of dramatic advances in neurosurgery, chemotherapies, and radiotherapies, the prognosis for glioblastoma multiforme remains unchanged and dismal, with a median survival time at diagnosis of 8–12 months.

### Biomechanics and mechanobiology

**Fig. 12:**
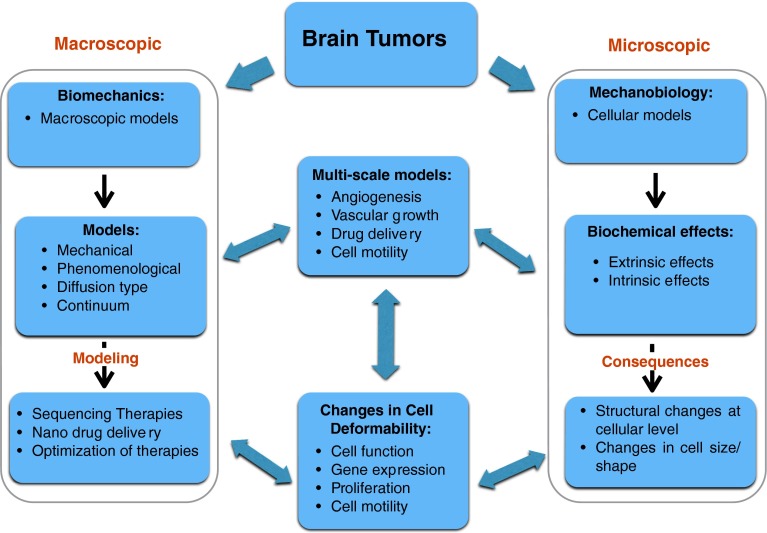
Schematic of modeling approaches to brain tumor growth and control at microscopic and macroscopic levels

Although the study of the structure and function of biological systems through the methods of mechanics is encompassed by the field of biomechanics, research in the biomechanics of tumors has focused at the macroscopic level on the determination of the mechanical properties of tissue-tumor systems, as summarized in Fig. 12. In contrast, the emerging field of mechanobiology, which lies at the interface of biology, engineering, and mechanics, has focused more on the manner in which physical forces act at the microscopic level. Thus, a central challenge in the mechanobiology of tumors is understanding the effects of mechanotransduction at the cellular level.

There has been an implicit assumption in medicine that the genesis and evolution of brain tumors—and cancers in general—have a genetic basis; however, progress in mechanobiology suggests that changes in cell mechanics, alterations in the microstructure of brain tissue, and cellular mechanotransduction may play an equally significant role in the development and progression of brain tumors. Brain tumors are phenotypically and genotypically heterogeneous and their complex interaction with the brain parenchyma is the major complication in existing therapies and in the development of appropriate new treatment strategies. Their diffusive, infiltrative nature makes brain tumors quite distinct from other solid tumors. In addition, the blood brain barrier is a major impediment to the effective delivery of chemotherapeutic agents. There is a real need to unravel and understand the cellular and molecular biology of glioblastoma multiforme, and these advances have to go hand in hand with both mathematical models and experimental progress. Although current medical imaging methods have become more sensitive and accurate in delineating anatomical features of the brain, they have not produced an effective early detection methodology. There has been significant progress on the biological front in identifying the molecular mechanisms of neoplasms in the brain, which has led to rapid identification of relevant molecular targets. The genomics and proteomics explosions suggest possibilities of developing high throughput approaches to determine gene function and thus suggest targets and pathways that form crucial components of brain tumor biology and function. However, advances in clinical oncology, neuroscience, cancer biology, and mathematical oncology make the present a propitious time for a multipronged attack on brain tumors. A collective, multi-disciplinary approach toward addressing many of the challenges of glioblastoma multiforme promises to deliver new diagnostic and therapeutic techniques that may hopefully lead to a cure for this deadly disease.

### Literature review

**Fig. 13:**
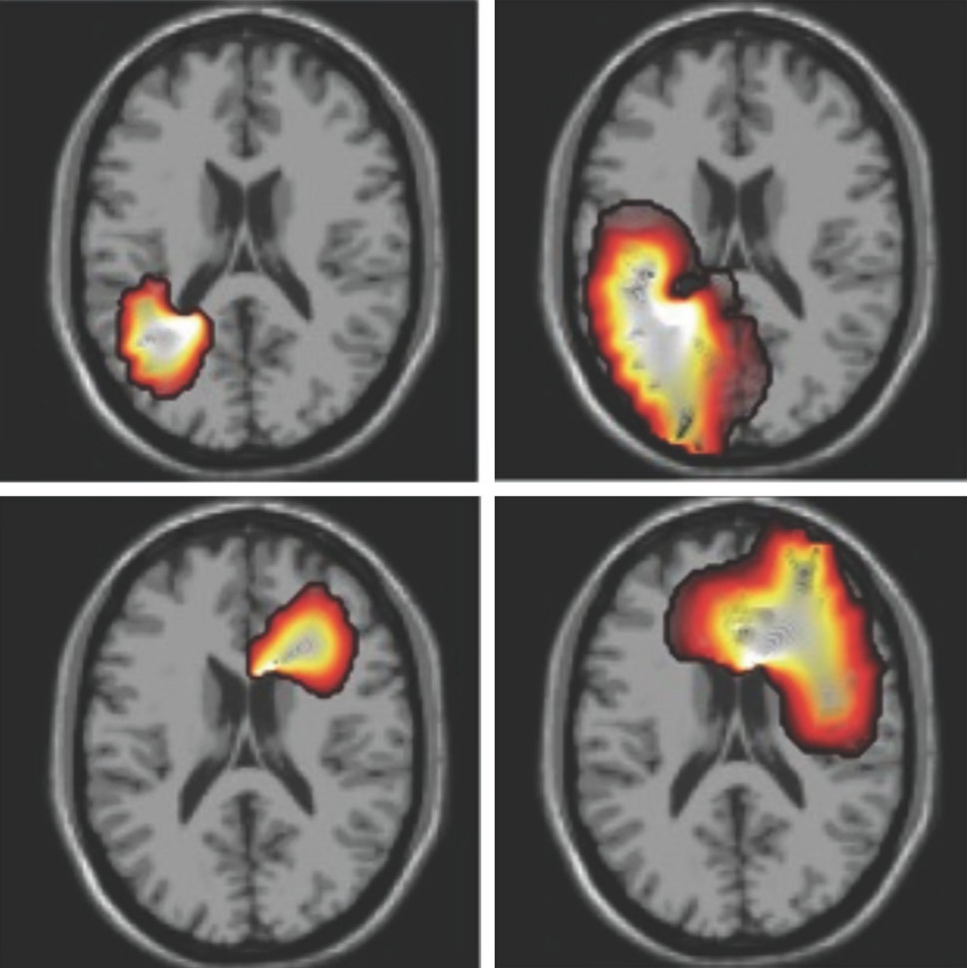
Simulation of glioblastoma multiforme at diagnosis and patient death, *left* and *right columns*. The *black contour* denotes the detectable tumor margin by computer tomography or magnetic resonance imaging. *Colors* indicate the gradient of malignant cell density with highest values at the center of the tumor and decreasing values toward the surrounding normal brain tissue, simulations by Gibin Powathil, University of Dundee

Radiotherapy is one of the primary therapeutic modalities for many primary and metastatic brain tumors. The gold standard to treat glioblastoma multiformes and other primary astrocytomas, still appears to be optimal surgical resection followed by radiotherapy. Despite significant progress, nearly all glioblastoma multiforme patients see tumor recurrence within the volume of tissue treated with high dose radiation and have a median survival time of approximately 12 months. Figure 13 shows the results of a computational simulation of glioblastoma multiforme growth and progression at diagnosis, left column, and at patient death, right column, and illustrates the potential of mathematical and computational modeling to assist therapeutic intervention.

The seminal contribution in modeling the effects of chemotherapy on glioblastoma multiforme was made by Murray and coworkers (Tracqui et al. 1995), who used a reaction-diffusion equation to model tumor growth and included a term modeling the cell kill kinetics of a chemotherapeutic agent. In subsequent work (Swanson et al. 2003), they quantified glioma growth and invasion and showed that these types of models could indeed be used as predictive tools in clinical oncology. Later work modeled tumor growth using a Fisher-type equation, incorporated the effects of radiotherapy through the linear quadratic model, and studied combination therapies of surgery, chemo- and radiotherapies (Powathil et al. 2007; Rockne et al. 2009). Tumor growth has also been studied through the use of discrete cellular automata models (Kansal et al. 2000). Cell metabolism can also be incorporated into these models through additional coupled partial differential equations, and the growth of the tumor at any particular location would be determined by the local availability of nutrients. These types of models have also been used to study the Warburg effect, the shift from aerobic respiration to glycolysis in cancer cells. The role of glycolysis in glioma progression and invasion has been studied using evolutionary game theory (Basanta et al. 2008). Studying the evolution of three different phenotypes under different microenvironmental conditions revealed that the invasive phenotype is selected preferentially under the glycolytic metabolism.

Evolutionary models have also been used to study gene expression and predict the sequence of somatic genetic events in the progression to cancer (Attolini et al. 2010). Pharmacokinetic and pharmacodynamic methods—particularly linear system analysis—have been used to predict the distribution of the drug temozolomide in tumors and normal tissue with particular reference to glioma patients (Rosso et al. 2009). The effects of radiotherapy on glioblastomas were investigated by assuming the existence of two cell populations: cancer stem cells and normal cells (Leder et al. 2014). Under the assumption that the cell kill rate differs for these two cell types and that radiation may induce normal cells to dedifferentiate into cancer stem cells, a linear quadratic model is used to study the effects of radiation on these cell types and the results are compared with the experimental data.

Finally, one of the more novel therapies for the treatment of gliomas is the use of alloreactive cytotoxic T lymphocytes in treating gliomas (Kronik et al. 2008). Interestingly, cell lines derived from human glioblastoma multiforme do not exhibit any significant radioresistance and it appears that the central nervous system environment and the tumor microenvironment play crucial roles in the radioresistance of glioblastoma multiforme. The field of radiation therapy has progressed significantly driven by technological advances together with new development in three-dimensional image-guided delivery systems. However, there is little encouraging sign that radiotherapy alone—even with dose escalation—has any greater impact on these intrinsically radioresistant cells. A more promising direction is the theoretical and experimental investigation of pharmacological agents that interact with radiotherapy synergistically.

### Open questions and challenges

The interactions between biomechanics, mechanobiology, and cancer biology, in particular cancers of the central nervous system, has recently given rise to a research area that has started to attract considerable interest across many disciplines, and it is a field that will undoubtedly gain increasing prominence in the coming years. Some of the major broad, open questions in the field, in which we can expect progress, include: What are the genetic pathways and mutations associated with oncogenesis that result in heterogeneity of brain tumors, and how can these be carefully examined and probed both experimentally and mathematically? How does the actual anatomical location of a tumor, together with its genetic profile, influence tumor heterogeneity and therapeutic efficacy? Which pathways, e.g., signaling, apoptosis, cell cycle, and migration, actively participate in brain tumor response to intracellular and extracellular stimuli, e.g., growth factors or redox changes?

Other questions relate to the modeling of the interaction between biological, biochemical processes, and the tumor microenvironment, and how these contribute to changes in the molecular structure of the cancer cell membrane and its cytoskeleton. It will be key to determine how the deformation characteristics and structural changes at the cellular level can be linked to the elastic and viscoelastic deformation characteristics at the macroscopic level. As a clearer understanding of the response of single cells to loading, as well as their mechanical properties emerges, how can these be generalized to a clonal mass through appropriate multi-scale modeling? In broader terms, how do the mechanical properties of tumor cells, alterations in their cell shape, and resultant variations in cell adhesion, affect the genesis and evolution of brain tumors. Finally, as the blood brain barrier of the central nervous system represents a major obstacle to drug delivery, how can biomechanical and mechanobiological models be used to study new treatment modalities?

### Concluding remarks

There is a critical need to develop appropriate models that will approximately replicate the biological complexity of our brain. These models can then be used to examine and probe interactions between brain tumor cells, cellular pathways, and constituents of normal brain function. At the same time, the models can be used to assess and appraise novel therapies that target precise genes and pathways. In these efforts, resources would be necessary to build up chemical and combinatorial libraries to investigate molecular targets and pathways. There is currently a dearth of mathematical models of immune system function in the central nervous system compartment. These types of models, coupled with current understanding of the cancer biology of the central nervous system, will be necessary to understand how brain tumors and the immune system interact in the central nervous system, especially when developing immunotherapies to treat glioblastoma multiforme and other types of brain tumors.

## Brain surgery and intracranial pressure

The intracranial pressure is the pressure inside the skull and is a function of the volume and compliance of each part of the various intracranial contents.

### Biomechanics and mechanobiology

In the normal homeostatic state, intracranial pressure is controlled closely by the body through a variety of mechanisms. However, this state can become impossible to maintain in pathological conditions. The intracranial pressure can decrease, or more frequently increase, with a consequent impact on brain function. Control of intracranial pressure is a vital aspect of treatment of patients with neurosurgical conditions and an understanding of intracranial pressure is important to allow us to understand the issues when thinking about modeling such a complex situation (Woods et al. 2009).

### Literature review

The most commonly used method of describing the interplay between intracranial components is the Monro–Kellie hypothesis proposed by a Scottish surgeon and his student in the late 18th century (Kellie 1824; Monro 1783). This method assumes a non-expansile fixed-volume skull (Mokri 2001). Therefore, the three main components within the skull–brain, cerebrospinal fluid, and blood—must exist in a state of volume equilibrium. This division in components implies that if one increases in volume, there needs to be a compensatory reduction in the other components. If this compensation does not occur, the intracranial pressure will rise. As an example, assume there is a rise in the volume of tissue, e.g., a swollen traumatized brain. The cerebrospinal fluid is first to exit the skull in compensation, followed by a reduction in volume of venous blood, and finally a restriction of arterial inflow.

If the rise is caused by an increase in cerebrospinal fluid volume, a reduction in volume of venous blood will occur (Jeevan et al. 2008); yet, there is also compression of the brain tissue itself. Of course, the skull is not a complete sphere and there is an exit hole through the foramen magnum through which the spinal cord exits, as well as various holes through which cranial nerves and vessels enter and exit the brain. Increasing the intracranial pressure leads to compression of important brain regions, and a risk of herniation syndromes, whereby certain regions may become pushed through these holes, stretched or compressed, which critically affects their function. In particular at the foramen magnum, dysfunction of the brainstem can lead to decreased consciousness, abnormal breathing, altered blood pressure and heart rate, coma, and finally death.

In a vicious cycle, some of these early symptoms can cause a further rise in intracranial pressure, in particular if breathing and oxygenation or removal of carbon dioxide from the blood are affected. Emergency neurosurgical treatment is designed around controlling and treating raised intracranial pressure and minimizing the effect of herniation syndromes on the function of the brain.

Reducing raised intracranial pressure allows maintenance of cerebral perfusion. The cerebral perfusion pressure is the difference between the mean arterial pressure and the intracranial pressure. Cerebral perfusion pressure is maintained by a mechanism of autoregulation wherein the brain’s arteries and arterioles are able to vary their overall diameter to maintain cerebral perfusion pressure when mean arterial pressure is between approximately 65 mmHg and 150 mmHg. Outside of these limits, however, autoregulation fails, with often disastrous consequences for the patient. A drop in systemic blood pressure, e.g., directly leads to a decrease in perfusion of the brain.

Intracranial pressure varies according to position and age. Pressure is higher when recumbent than when upright. When lying down, the pressure will also rise when the patient is asleep, in particular due to retention of carbon dioxide during sleep. The positional aspect is in particular related to venous drainage and cerebrospinal fluid flow into the spinal column being assisted by gravity. The intracranial pressure will also vary according to age with newborns having an intracranial pressure of roughly 0 mmHg. Babies have an intracranial pressure of between 5 and 10 mmHg, children have a supine intracranial pressure of approximately 12 mmHg, and adults have an intracranial pressure up to approximately 10 mmHg (Morritt et al. 2010).

When measuring the intracranial pressure, the seminal work by Lundberg in the 1960s showed three waveform patterns, A-waves, B-waves, and C-waves (Lundberg et al. 1965; Risberg et al. 1969). C-waves are the normal variation in intracranial pressure with the cardiac cycle. B-waves occur during periods of moderate intracranial pressure elevation up to 20-40 mmHg, lasting between 5 and 10 minutes, which then return back to their normal baseline. They can be seen in particular during the sleep without any pathological importance. Three B-waves within a 24-hour period can be accepted as within normal limits (Wall et al. 2014). A-waves, which are also called plateaus, are very abnormal waveforms where the pressure will suddenly rise to often above 50 mmHg and last 30 minutes or more; once the waveform starts to return to normal, it often does not fully return back to the original baseline. A-waves are indicative of a loss of compliance of brain tissue and autoregulation of cerebral blood flow. They occur in the acute phase of impending catastrophic intracranial pressure waves with herniation and are a very poor prognostic sign.

**Fig. 14:**
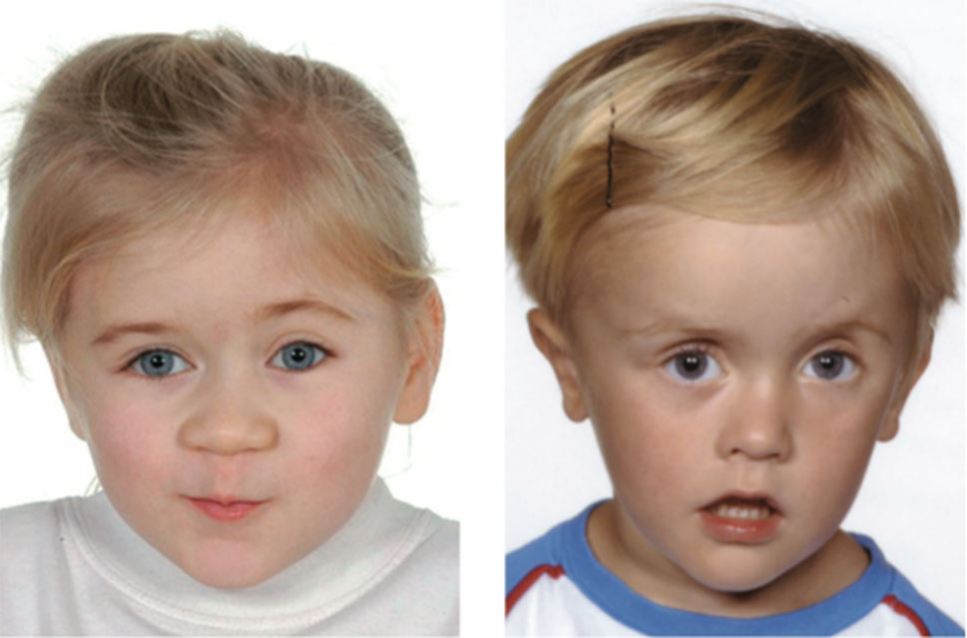
Preoperative appearance of children with unicoronal craniosynostosis, an early fusion of the skull plates. Craniosynostosis causes an increase in intracranial pressure and requires intracranial pressure monitoring, adapted from Eley et al. (2012)

**Fig. 15:**
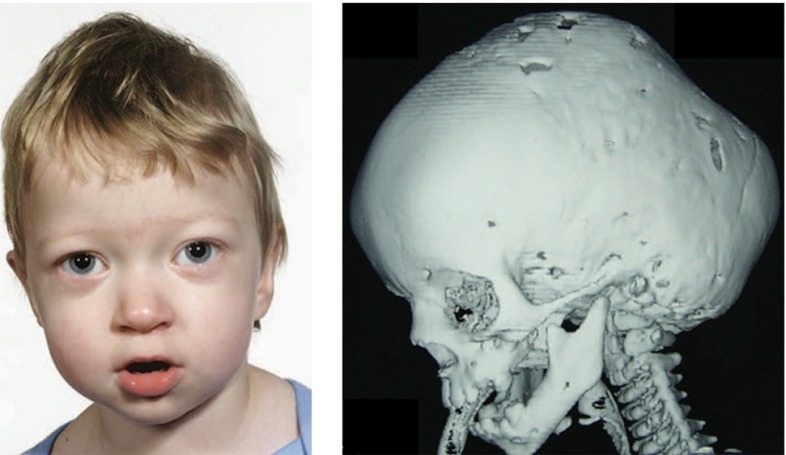
Clinical photograph and computed tomographic scan of a pediatric patient a year after modified strip craniectomy for sagittal synostosis, demonstrating vertex bulge and bicoronal synostosis (Marucci et al. 2008)

When looking at the complex brain in the young, there are some additional difficulties. A baby’s skull is not a fixed volume. The sutures, which allow for growth between the different plates of the skull also allow for some expansion of skull volume as compensation for raised intracranial volume. In particular chronic conditions, slow rises may be accommodated by an expansion of the head size, rather than by an increase in intracranial pressure. For instance, patients with craniosynostosis, an early fusion of the skull plates illustrated in Fig. 14, show raised intracranial pressure (Jeevan et al. 2008). Strip craniectomy, a surgical operation that removes part of the skull to allow for further expansion, reduces the intracranial pressure, but may lead to bulging (Marucci et al. 2008) as illustrated in Fig. 15. Further monitoring of the intracranial pressure is critical to ensure normal development (Eley et al. 2012).

### Open questions and challenges

The complex relationship between skull volume and pressure implies that the Monro–Kellie hypothesis does not apply under pathological conditions. Also, in young populations, the precise importance of levels of intracranial pressure and cerebral perfusion pressure are not fully understood. A cerebral perfusion pressure of 60 or 65 mmHg will often be taken by intensivists and neurosurgeons as the aim of any treatment. However, there is no really strong evidence to support this value as an absolute target. Since treatment may involve drugs, which have significant effects on blood pressure throughout the body and other side effects, the need to chase a number on a screen must always be balanced by potential side effects of such treatment. Sometimes a lower cerebral perfusion pressure may be preferred over the side effects that can occur with targeted therapy.

Clinically trying to examine the outcome of patients who have raised intracranial pressure is often difficult. Rarely do these patients have solely a raised intracranial pressure without, e.g., traumatic injuries to the brain or developmental abnormalities such as neonatal hydrocephalus. Being able to extricate those long-term issues caused purely by the raised intracranial pressure, rather than by these parallel conditions, becomes difficult. To date, there is a severe lack of sufficiently large patient population data to make firm statements about outcomes with reliable statistical strength.

### Concluding remarks

Intracranial pressure and its assessment are important tools to understand both brain modeling and clinical issues when deciding treatment algorithms for individual patients. For modeling to be successful we will need a significant increase in our level of understanding of the influence of intracranial pressure on brain function, and equally, the potential for brain function to affect intracranial pressure. In particular, the constant changes of intracranial pressure and autoregulation will be difficult to model, especially since we do not fully understand all the mechanisms that control intracranial pressure in both children and adults. It is only by a collaborative approach between clinician scientists and mathematical modelers that we will move closer to understanding this intricate situation.

## Traumatic brain injury

### Biomechanics and mechanobiology

**Fig. 16:**
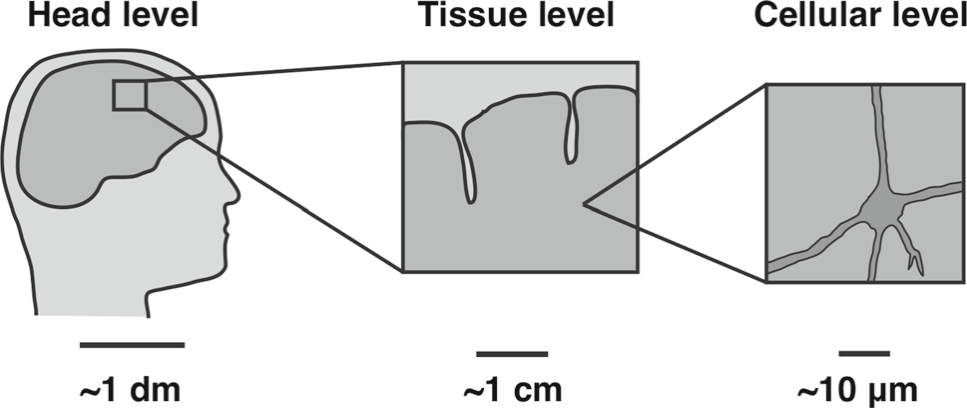
The length scales involved with traumatic brain injury ranging from decimeters at the head level to micrometers at the cellular level, reproduced from Cloots et al. (2012)

Traumatic brain injury causes high rates of mortality and disability (Brooks et al. 1997; Waxweiler et al. 1995) and results from a mechanical insult on the head, often due to traffic accidents but also due to sports accidents and falls (Jennett 1996). As discussed in Sects. 4 and 5, the mechanical impact on the head is translated into deformation of the brain tissue, which sets in motion an extended neurochemical cascade on the cellular level (Gennarelli 1993; Morrison et al. 1998). Thereby, the mechanism of traumatic brain injury involves the biomechanics and mechanobiology at various length scales (Cloots et al. 2011, 2013; Meaney et al. 2014), see Fig. 16.

### Literature review

The most commonly used brain injury criterion in the automotive industry is the Head Injury Criterion, a scalar function of the translational acceleration pulse of the head (NHTSA 1972), which is used for safety assessments based on the interpretation of crash tests and virtual experiments. The Head Injury Criterion is quantified using experimental data, in which only anterior–posterior contact loading has been applied to human cadavers. One of the major points of criticism of the Head Injury Criterion is that it does not account for angular accelerations, which have been shown to play a significant role during brain motion in vivo (Ibrahim et al. 2010; Sabet et al. 2008). Furthermore, the Head Injury Criterion does not distinguish between different injury mechanisms. For a better understanding of the relation between a mechanical load and traumatic brain injury, more advanced methods have been developed in which the internal head response is considered. These methods include the usage of physical and numerical models of the head and the brain (Margulies et al. 1998; Ivarsson et al. 2000).

**Fig. 17:**
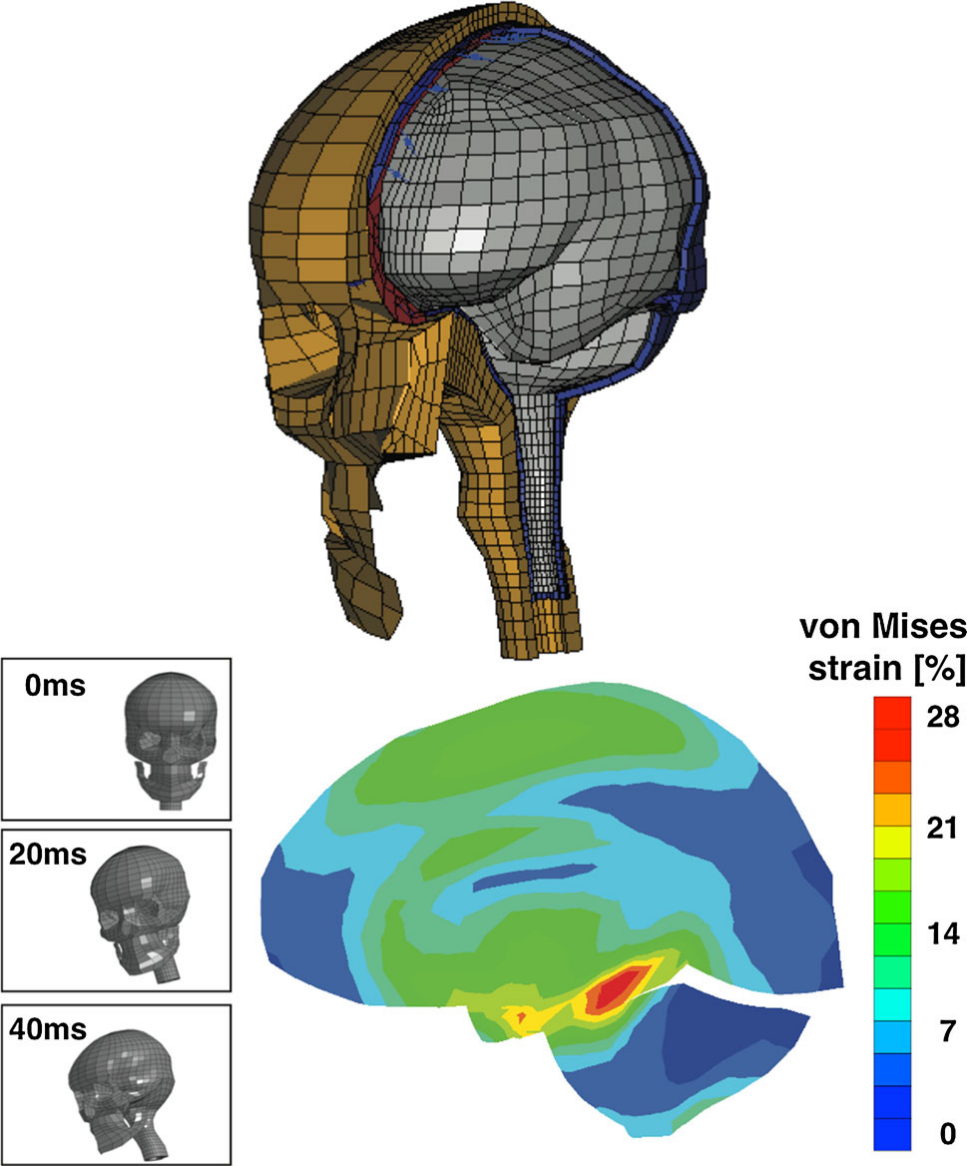
Finite element head model of Kleiven 2007 and predicted maximum principal strain in a sports accident, reproduced from Cloots et al. (2013)

Numerical models to predict head injury were initially developed in the 1970s and early 1980s, but more recent numerical head models were not developed until the early 1990s (Goldsmith 2001). Since then, numerical head models were continuously refined to include more details of the anatomy and the mechanical behavior of the head and brain. In the past decade, three-dimensional numerical head models have been developed, containing viscoelastic material behavior and internal geometries that account for the main anatomical substructures including the ventricles and the falces (Kleiven 2007; Hrapko et al. 2009), see Fig. 17. Validation of head models often relies on the results of a number of cadaver experiments (Nahum et al. 1977; Hardy et al. 2001). The morphological heterogeneities of the cerebral cortex in the form of gyri and sulci are important for the deformation that is experienced by the tissue (Cloots et al. 2008). In the latest head models, more and more of these heterogeneities are incorporated. For example, the most recent three-dimensional head models even contain the detailed substructures of the cerebral cortex (Ho and Kleiven 2009; Chen and Ostoja-Starzewski 2010).

Besides a detailed description of anatomical structures, the interaction between the various components inside the skull plays a crucial role. In particular, the cerebrospinal fluid is considered to have a protective function for the brain during mechanical loading, making it an important constituent of the head with respect to traumatic brain injury. The cerebrospinal fluid and the meningeal layers are often modeled either as a solid layer with a low shear modulus or as a sliding interface

**Fig. 18:**
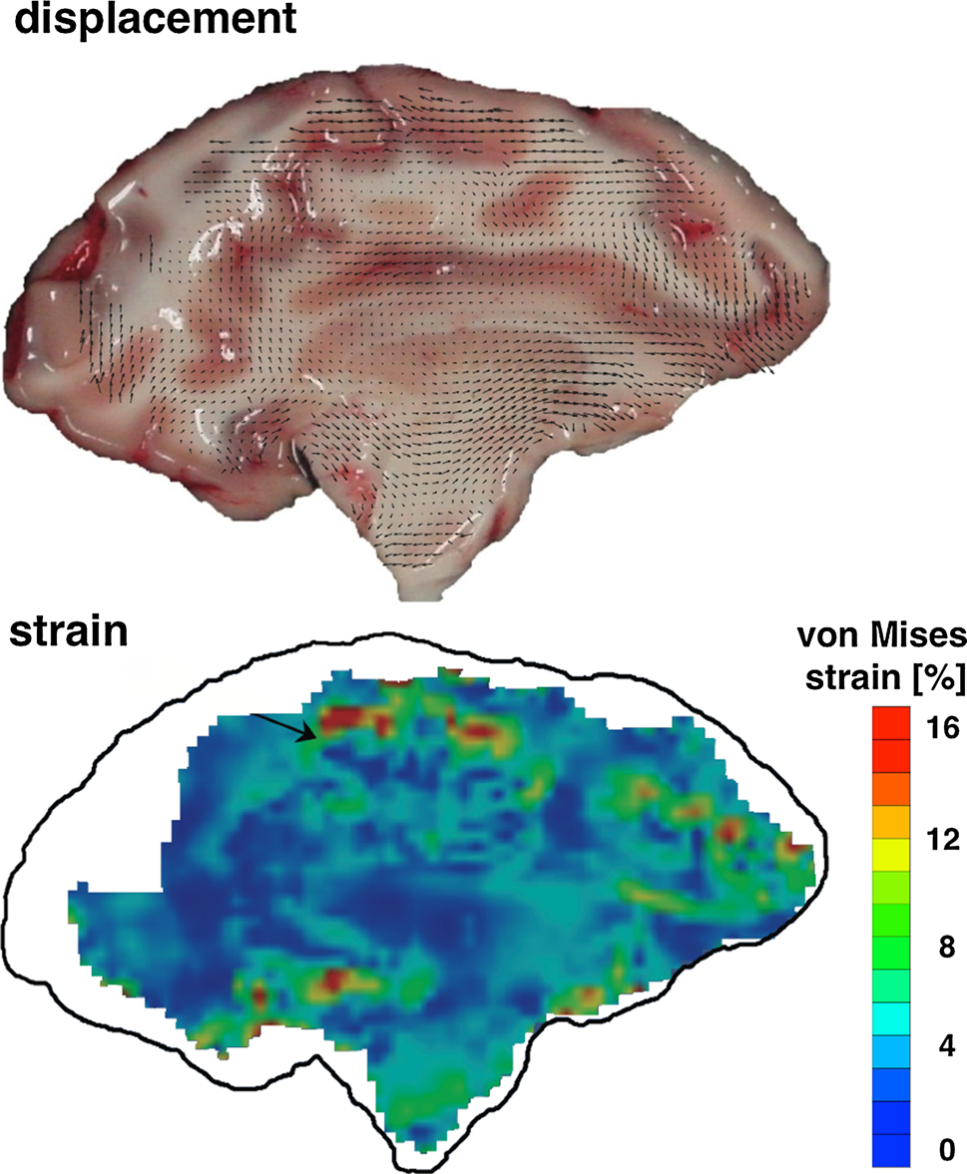
Local displacement field in an accelerated porcine brain slice and corresponding equivalent von Mises strain field, reproduced from Lauret et al. (2009)

In both in vivo and in vitro experimental studies, the complex deformation of the brain during an impact load has been measured. For example, brain deformation during mild acceleration of the head of volunteers has been quantified using magnetic resonance imaging (Bayly et al. 2005; Feng et al. 2010), helping to elucidate the role of the skull–brain interface and the mechanisms leading to traumatic brain injury. In another study (Lauret et al. 2009), full-field deformation data were obtained from digital image correlation on slices of porcine brain tissue subjected to higher accelerations, showing the complexity of the deformation field and the role of the substructure of the brain, see Fig. 18. These experimental investigations, where full-field deformation with a high spatial resolution is obtained, may enable a further, more detailed, validation of numerical head models (Ibrahim et al. 2010; Sabet et al. 2008).

For reliable prediction of the mechanical response of the brain with numerical head models, an accurate description of the constitutive behavior of its constituents is required. Various approaches to characterize and model the viscoelastic response of brain tissue exist (Van Dommelen et al. 2011). A constitutive law for a numerical head model should accurately describe the mechanical response of brain tissue for large deformations, complex loading histories, high strain rates—the loading typically changes within milliseconds—and deformation modes that occur during an impact to the head. Although some accurate nonlinear constitutive models have been proposed (Hrapko et al. 2006; Bilston et al. 2001), most head models are still based on a linear viscoelastic description (Hrapko et al. 2009).

As discussed in Sect. 2, methods to characterize the mechanical properties of brain tissue include rheological approaches based on shear or compressive loading. With these methods, the viscoelastic properties of brain tissue have been characterized, leading to a wide variety of data, however, with also a large variation between studies (Hrapko et al. 2008). Several recent studies have employed indentation techniques to investigate local variations in mechanical properties of brain tissue (Budday et al. 2015b; Dommelen et al. 2010; Elkin et al. 2011), which is required as numerical models of the head become more and more detailed. In particular, a difference between the properties of white and gray matter has been demonstrated. A potentially powerful technique that has seen much progress recently is magnetic resonance elastography (Sack et al. 2008; Hamhaber et al. 2010; Sack et al. 2011; Romano et al. 2012), a method that enables in vivo measurements of the local tissue stiffness at small strains with a high spatial resolution (Green et al. 2008; Vappou et al. 2007).

As mechanical properties of brain tissue are becoming more accurately characterized, the pronounced anisotropy of brain tissue becomes more apparent (Prange and Margulies 2002; Hrapko et al. 2008); especially, white matter has been observed to be anisotropic due to its oriented axonal tracts, both in tension and shear (Feng et al. 2013; Velardi et al. 2006). A fiber-based constitutive model for brain tissue was used in (Cloots et al. 2011) in a tissue-level numerical analysis of traumatic brain injury and in (Cloots et al. 2013) in a multi-scale analysis. In recent studies, anisotropy of brain tissue was incorporated in full-scale numerical head models for traumatic brain injury to demonstrate the importance of orientation-dependent mechanical properties of the tissue (Giordano et al. 2014; Wright et al. 2013). In these studies, diffusion tensor imaging was used to identify spatially resolved axonal directions and the degree of axonal orientation.

Numerical head models form a bridge from a macroscopic load on the head toward the fine scale loads at the tissue level. While the classical Heard Injury Criterion provides a criterion for injury at a global kinematic level, the use of numerical head models necessitates novel injury criteria at the level of the brain tissue, which are often based on equivalent stress, strain, or pressure. Quantitative criteria for injury of brain tissue have been obtained through accident reconstructions (Kleiven 2007; Deck and Willinger 2008), where predictions of tissue-level mechanical fields for real-world accidents are matched to the actual clinical outcome of these accidents and via in vitro models (Morrison et al. 2006), where organotypic brain slice cultures stretched on a membrane were used. In addition to anisotropic constitutive properties, the oriented axonal structure of white matter can also be expected to lead to an orientation-dependent sensitivity of brain tissue to mechanical loads with respect to injury. Injury thresholds at the axonal level have been established using the optic nerve of the guinea pig (Bain et al. 2001). Based on axonal strain, an anisotropic tissue-level injury criterion was developed, where the orientation dependence is based on the local underlying axonal directions (Cloots et al. 2012). This criterion was recently evaluated using multi-scale simulations (Cloots et al. 2013).

### Open questions and challenges

There remains a critical need for an accurate injury predictor that is applicable for all loading conditions and is able to distinguish between different injury mechanisms, but at the same time is also robust and efficient. To convert numerical head models into reliable and accurate injury predictors, a sufficiently geometrically detailed description of the various anatomical structures is required, as well as a precise characterization of their interactions such as the skull–brain interface. The constitutive model used for the various structures should be sufficiently accurate, in particular capturing the high strain rate behavior and possible nonlinearity of the tissue. The main challenge in modeling and characterizing the tissue response is to determine the regional variation and local anisotropy of the tissue. Since these aspects are directly related to the cellular structure of the tissue and variations thereof, a constitutive description based on the cellular morphology appears to be a physically realistic and feasible approach. In that case, spatial variations in properties are described in terms of morphological variations.

On the one hand, numerical head models may become more detailed. On the other hand, the accuracy of their validation procedures should be improved strongly. Most current validations rely on spatially coarse measurements of local mechanical quantities such as pressure or displacement. For more reliable validations, high resolution in vivo or in situ strain measurements should be used, although the general acceptance of such detailed datasets for this purpose remains a concern.

By addressing these challenges, the predictions of mechanical fields such as stress or strain should become more reliable and therefore more predictive. In order to extent these predictive capabilities toward injury, spatially varying, truly local and orientation-dependent injury criteria are required. Since the sensitivity of the brain tissue to mechanical loads depends on the cellular morphology as well, local injury criteria should also rely on the underlying microstructure of that local tissue. More specifically, if reliable mechanical predictions can be achieved at the level of axonal stretching, a threshold for injury can be specified at this level. This knowledge will allow us to establish truly mechanistic, local tissue-level injury criteria. Besides these mechanical aspects of traumatic brain injury, many studies have focused on the physiological processes of neurological injury. To fully understand the process of injury development in the brain at all length scales involved, these fields should be brought together.

By incorporating these subject-specific geometric and morphological details, numerical head models become increasingly more complex and computationally demanding. At the same time, these models should remain robust and computationally efficient, which is an obvious contradiction. Therefore, the development of a computationally efficient numerical model with high predictive capabilities remains a major challenge. Finally, to benefit from these developments, novel injury assessment methods based on numerical simulations will have to be acknowledged by the regulatory agencies and incorporated into injury regulations.

### Concluding remarks

Computational multi-scale head models form a potentially powerful next generation of injury assessment tools. They involve the mechanics of brain tissue specifically in the high strain rate regime. In a multi-scale approach, they can address the various length scales involved in traumatic brain injury. The morphologic dependence of mechanical properties extends to applications in lower strain rate regimes, e.g., during medical interventions and development of neurological disorders. To a lesser extent, this approach also may hold for structure-based injury criteria. Although originally developed to assess brain injury, numerical head models may serve other purposes leading to further understanding of the mechanics and functionality of the brain.

## Shaken baby syndrome

Closely related to traumatic brain injuries, shaken baby syndrome is widely accepted as a form of child abuse, defined by a triad of intracranial findings: retinal bleeding, subdural bleeding, and encephalopathy. Many of the syndromes and pathologies also apply to the previous section. However, due to the specific and unique features of young brains, the following section is dedicated to their cause and effect in babies. The notion of shaken baby syndrome arose from early descriptions of subdural hemorrhage in association with multiple skeletal injuries and bruises in babies thought to have been abused (Caffey 1972). Whiplash during shaking was hypothesized to cause the characteristic bilateral thin film subdural hemorrhage (Guthkelch 1971). This hypothesis rapidly gained popular support, was incorporated into mainstream pediatrics, and is now applied to infants with the triad even if they have no other evidence or history of trauma. Alternative names such as abusive head trauma and lethal craniocerebral trauma have been suggested (Christian and Block 2009), but are inappropriate because they imply intent such as abuse, or a mechanism such as trauma, which is not the only cause of the findings. Instead, the focus should be shifted to the objective pathology underlying these three features and the term retinodural hemorrhage of infancy is suggested as a more accurate description of the findings. Here, we discuss the pathology of each of the features and review their mechanical origin.

### Biomechanics and mechanobiology

Several biomechanical concepts have been incorporated into the shaken baby hypothesis. Rotational acceleration has long been recognized as the main cause of diffuse traumatic injury in the brain. The concept that shaking causes subdural bleeding by the mechanism of bridging vein rupture was introduced in the early 1970s (Guthkelch 1971). However, experiments on infant models showed that the angular acceleration and velocity produced by shaking fall far below the calculated injury range, and were smaller by a factor of 50 than those generated by impact (Duhaime et al. 1987). Nonetheless, proponents of the shaken baby hypothesis continue to maintain that “The application of rotational acceleration and deceleration forces to the infant’s head causes the brain to rotate in the skull. Abrupt deceleration allows continuing brain rotation until bridging veins are stretched and ruptured, causing a thin layer of subdural hemorrhage on the surface of the brain” (Harding et al. 2004).

### General description

The pathophysiology of human traumatic brain injury is highly complex; the most important first principle is the recognition that primary mechanical damage may be followed by secondary brain changes in response to trauma. Trauma causes primary mechanical deformation of the brain, followed by a complex secondary cascade of events including brain swelling, changes in brain perfusion, hemorrhage, release of neurotransmitters, neurogenic inflammation, and cortical spreading depression (Lauritzen et al. 2011). The importance of recognizing this principle is threefold: Firstly, the secondary mechanisms need to be understood as some may be amenable to therapy; secondly, the clinical manifestations of head trauma may not be seen immediately, but may develop only as the secondary cascade evolves, and these patients may suffer a lucid interval, that is, have a period after trauma with no symptoms or mild, nonspecific, symptoms before developing severe symptoms related to the secondary cascade; thirdly, the secondary cascade effects are not unique to trauma, and therefore, the identification of nonspecific signs of brain dysfunction such as seizures or edema cannot automatically lead to the assumption that trauma has taken place. The three features of the triad are closely interrelated responses to alteration of intracranial homeostasis and have many causes.

*Retinal Bleeding.* Retinal hemorrhages have been regarded as an important indicator of inflicted injury, but there are many other causes of retinal bleeding in infants, including normal birth, raised intracranial pressure, and intracranial hemorrhage (Bhardwaj et al. 2010). Two main mechanisms are proposed: The first is venous outflow obstruction in raised intracranial or intravascular pressure; the second is mechanical, commonly referred to as vitreo-retinal traction (Wygnanski-Jaffe et al. 2009). Pathological studies indicate that venous stasis and leakage from retinal vessels are a more likely cause of retinal bleeding than vitreo-retinal traction (Emerson et al. 2007). The compromised venous outflow hypothesis is also supported by the observation that, when asymmetric, retinal hemorrhages predominate on the side of greater brain injury (Gilles et al. 2003). Further, there is a statistically significant relationship between retinal and optic nerve sheath hemorrhage and reperfusion, cardiopulmonary resuscitation, and cerebral edema (Matshes 2010).

**Fig. 19:**
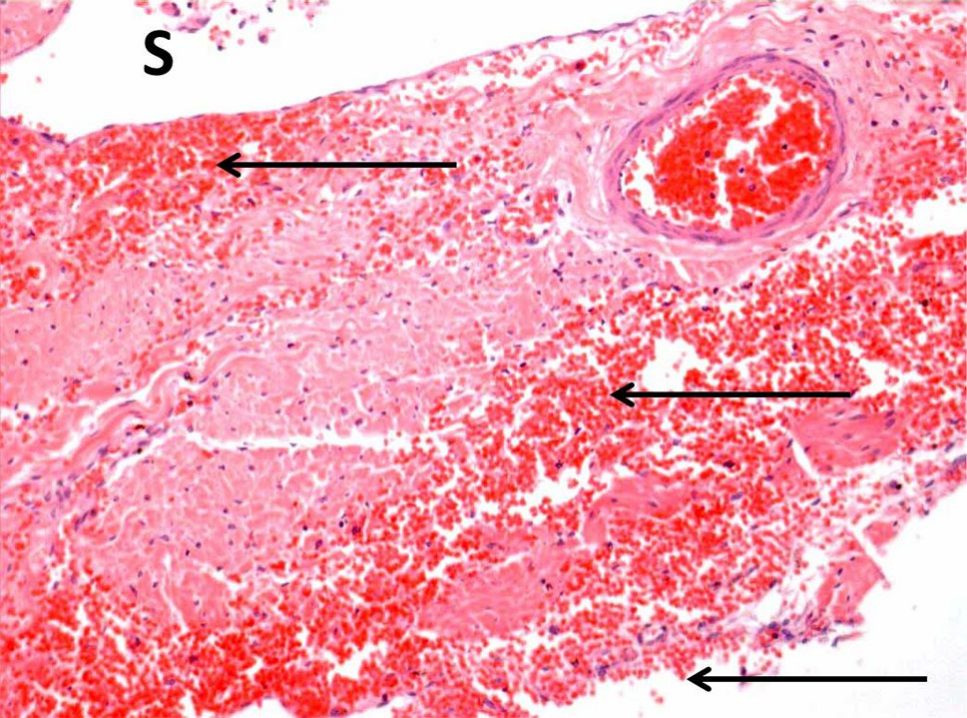
Dural bleeding. Collagen fibers of the dura are stained pink; blood cells are stained red. *Arrows* indicate hemorrhage around the venous sinus ($S$), between the fibers of the dura, and extending onto the deep surface

*Dural Bleeding.* The typical pattern of subdural hemorrhage in babies with the triad is of a bilateral thin film over the cerebral convexities and in the posterior interhemispheric fissure (Duhaime et al. 1998), a distribution seen also in a proportion of healthy newborn infants and corresponding to the most extensively vascularized areas of the dura (Tubbs et al. 2007). Because in vivo dura is adherent to the underlying arachnoid membrane (Haines et al. 1993), all dural bleeding is, by definition, intradural. When dural bleeding is sufficiently extensive, it cleaves the deepest dural cell layers, creating a subdural compartment. At autopsy, the clot will be observed within and beneath the native dura and above the arachnoid membrane, see Fig. 19.

Radiologically, however, it may be impossible to determine whether or not a small dural collection is entirely intradural or has cleaved the dural border layer and become subdural (Squier 2011). The original shaking hypothesis suggested that shaking ruptures the bridging veins, which carry blood from the cortex of the brain into the venous sinuses within the dura. These veins carry large volumes of blood, which would produce large volume, space-occupying clots rather than the thin film bleeds typical of shaken baby syndrome. The explanation for extensive, thin dural bleeds in infants may be found in the age-specific pathophysiology of the dural vasculature in infants. Intradural and subdural bleeding is almost universal in neonates; even in those who lack evidence of either trauma or overt hypoxia (Gilles and Nelson 2012).

**Fig. 20:**
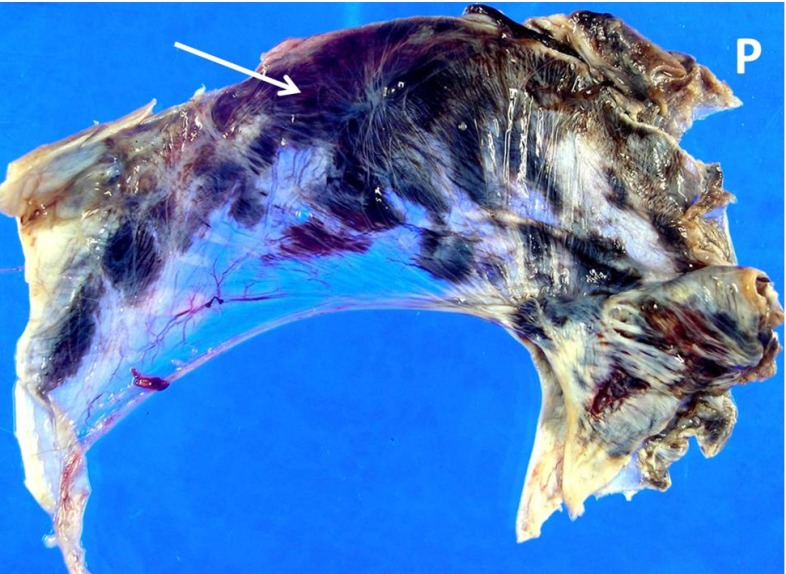
Dural bleeding. Fresh bleeding into the falx. *White arrows* indicate blood adjacent to the superior sagittal sinus and in the posterior falx ($P$)

Dural venous congestion and bleeding may represent a normal phenomenon and protect the brain during birth. The dural venous plexuses, which are the likely source of this bleeding, are larger and more complex at birth than at any time in later life (Mack et al. 2009), see Fig. 20. Venous blood flows from the dural sinuses via valveless neck veins to return to the heart. Studies in newborns have shown that pressure on the head, altered head position, obstruction of the jugular veins and mechanical positive pressure ventilation can all increase the pressure within the dural sinuses. The pressures exerted on the infant during labor and delivery may similarly compromise venous return to the heart and increase dural venous pressure. Were these pressures to be transmitted to veins in the brain they would bleed, but sphincters at the outflow cuffs of the bridging veins may prevent such back pressure (Chen et al. 2012). Since there are no sphincters on the small dural vessels (Squier et al. 2009), increased pressure in the dural sinuses may lead to reflux with venous congestion and bleeding into the dura which, if extensive, can leak into the subdural compartment and appear as subdural bleeding. The large venous lakes in the immature dura lesions have been assumed to represent reservoirs, additional protection against reflux of venous blood into the brain during the considerable pressure fluxes to which it is exposed at birth.

*Encephalopathy.* Encephalopathy is a vague concept in the context of infant brain injury and may represent any kind of brain malfunction from lethargy and vomiting to severe fulminating brain swelling. Originally, it was thought to be due to diffuse traumatic axonal injury but this pathology was shown to be rare; most babies thought to have suffered non-accidental head injury had global hypoxic-ischemic injuries (Geddes et al. 2001). As radiologically identified lesions may represent traumatic axonal injury and imply the causes and mechanisms of head injury (Hymel et al. 2010), a close examination of what is known about axonal damage in infant head trauma is warranted.

**Fig. 21:**
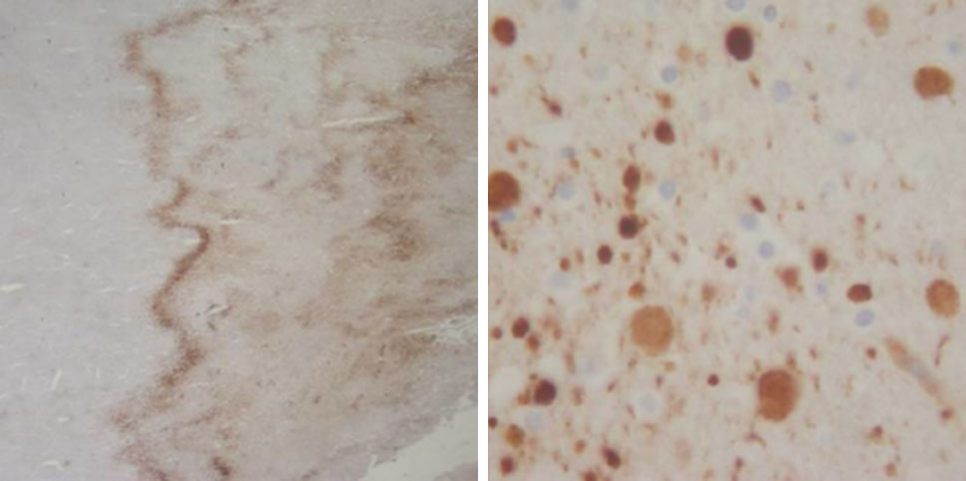
Axonal staining with beta-amyloid precursor protein ($\beta$-APP). At low power, a wavy pattern of staining seen in ischemic areas (*left*); at higher power, rounded profiles stain *brown*, these represent individual swollen axons (*right*)

*Axonal Injury.* Axonal injury can only be identified by microscopy and is demonstrated using the $\beta$-APP stain, which highlights swellings and varicosities in damaged nerve fibers, see Fig. 21. These swellings do not represent damaged ends of severed axons, but rather reflect healing changes in response to disrupted axonal transport. While they may result from mechanical axonal damage, they can also result from many other etiologies, including deprived blood or oxygen supply and metabolic disturbance. Stretch injury leads to ultrastructural changes within the axon, both proximal and distal to the injury site, which may evolve for days after injury. While some changes are reversible, some may lead to permanent secondary axotomy (Maxwell et al. 2003).

In the adult brain, diffuse axonal injury may be associated with focal lesions, typically seen in the deep white matter structures. These are usually, but not inevitably, hemorrhagic but are characteristically surrounded by axonal swelling and large amounts of $\beta$-APP (Graham et al. 2000). The pathology of axonal injury in infants is quite distinct and the patterns differ from those in adults (Reichard et al. 2003). In a study of 18 infants assumed to have been shaken, identification of focal axonal injury was not reliable after a certain survival time and discrimination between traumatic and ischemic axonal injury was not possible in every case, because the phenomena appeared to be of combined origin (Oehmichen et al. 2008). The axonal injury in another study was in a very specific distribution: It was not associated with hemorrhage suggesting that localized traumatic axonal injury at the craniocervical junction was due to non-disruptive stretch injury to the neuraxis (Geddes et al. 2001). This hypothesis rests on the assumption that axons are more sensitive to mechanical stretching than their associated small blood vessels. Radiologically identified hemorrhage is not a surrogate for axonal injury. An animal model of rotational head injury revealed that increasing rotational acceleration leads to greater axonal injury, but there was also more post-injury apnea and more ischemic damage (Ibrahim and Margulies 2010). This questions the reliability of axonal damage as identified by $\beta$-APP in the infant brain as a marker of trauma. Changes may be due to ischemia or secondary phenomena and, while axonal injury may follow trauma, it is neither specific for, nor diagnostic of, mechanical damage.

*Subcortical Contusions.* Contusions of the mature brain are superficial, involving the cortex. They are hemorrhagic, later developing into shrunken brown scars. The term contusion, usually used as synonymous with bruising, implies mechanical tissue damage and includes bleeding from small blood vessels. Surface contusion is extremely rare in the infant brain. A quite distinct pathology, typically seen in infants in the first 5 months of life has been labeled subcortical contusion, a term which has led to a great deal of confusion. The original report of subcortical contusions in infants who were under 5 months of age and thought to have been abused, described little or no necrosis and no axonal swellings around the clefts (Lindenberg and Freytag 1969). All of the babies who died shortly after injury had markedly swollen brains the pathology of their cysts was the same as that described in circulatory disorders in infants. Similar lesions were found in another study, but, again no axonal swellings were seen, nor any associated hemorrhage; an important distinction from adult diffuse axonal injury (Calder et al. 1984). These pathological studies suggest that subcortical contusions are not specific to trauma and may result from ischemia and brain swelling (Squier 2011). There is no pathological support for the hypothesis that subcortical contusions are the result of the cortex sliding over the white matter during shaking (Jaspan et al. 1992).

*The Secondary Cascade.* The secondary cascade of events in infant brain trauma remains poorly understood and demands further study in view of the potential for intervention and therapy. Brain swelling and altered perfusion may be mediated by autonomic and trigeminal neurovascular responses and modified by neurogenic inflammation and cortical spreading depression (Lauritzen et al. 2011). Trigeminovascular responses may be sensitized by prior episodes of trauma, subdural bleeding, or inflammation. This effect could augment cerebrovascular responses, explaining phenomena such as the big black brain and second impact syndrome, typical seen in young patients with subdural bleeding (Squier et al. 2012).

### Concluding remarks

In the four decades since the shaken baby syndrome hypothesis was first proposed, clinical, pathological, and biomechanical studies have failed to reveal a scientific basis for it. There is mounting evidence that retinodural bleeding in infancy is not specific to trauma, but is rather the result of age-related vulnerabilities of the infant brain and dura. Mechanical modeling holds the potential to provide a better understanding of the underlying mechanisms of injury with the ultimate goal to classify and diagnose the conditions which lead to retinodural bleeding in infants

## Conclusions

The brain, the center of the nervous system, lives in a physical world and must abide by physical laws dictated by its composition and environment. In particular, mechanics and geometry play a central role in brain development as well as in normal and abnormal brain physiology. In this review, we analyzed the individual contributions of the solid, the fluid, the electromechanical, and the electrochemical phases separately. However, it should be clear that all these contributions conspire for a robust but very tight control of the shape, connection, and function of the brain. From a simple mechanical point of view, the brain offers formidable challenges emanating from the fact that its protective embedding is a hard skull, its tissue is extremely soft, interlaced with axons fibers and a fluid and blood network, and very sensitive to changes in its osmotic environment.

The first challenge is to obtain a reliable global model, or rather classes of models for the brain that allow to reliably predict deformation, strain, stress, and circulation. These models need to be properly validated and should be supplemented by a realistic geometry and appropriate loading and boundary conditions. For the particular phenomenon of traumatic brain injury, the field of mechanical modeling is already very advanced—for many other applications, the field is wide open. Ideally, a valuable mechanical model combines as many of the mechanical phases described in this review as necessary to fully characterize the underlying phenomena. In addition, it should include the coupling between the individual phases. This approach is crucial to model multi-field phenomena including edema formation, tumor growth, and brain surgery.

The second major challenge is to connect the multiple scales, which interact in our brain. A multi-scale framework that integrates biomechanics and mechanobiology is the key to link mechanics and function as well as geometry and connections. Ideally, our models should be designed such that the smaller molecular and cellular scales inform the larger tissue and organ scales. In this setup, global metrics of form and function follow naturally once the model is calibrated correctly. Bottom-up models allow us to draw conclusions about how mechanical events affect or disrupt cell level processes and organ level function and about how the brain organizes itself during development.

The ultimate challenge of the nascent field of brain mechanics is to connect mechanics to cognitive processes by integrating the latest development of neurosciences including geometric and topological methods from network theory with a fine description of the geometry and mechanics of the brain. This long-term goal would provide a rational basis to address fundamental mental disorders related to the geometry of folding and the topology of connections such as epilepsy and schizophrenia and to understand long-term psychological problems associated with brain injury, cancer, or dementia.

Successful modeling requires concerted efforts between mathematicians, biologists, physicists, engineers, physiologists, histologists, neuroscientists, neurologists, neurosurgeons, and psychologists. Finding a common language between disciplines is often a critical road block in such an endeavor, which we have tried to address here. We hope that this review will spark discussion and stimulate research in brain mechanics with the goal of providing a holistic understanding of the physiology and pathophysiology of our most complex but least understood organ.

## Glossary of biological terms

Axons: Long slender projection of neurons, that transmit information in the form of electrical signals to other neurons and cells in the body
Aqueduct of Sylvius: A canal that contains CSF and connects the third and fourth ventricle
Astrocytes: A type of glial cell cells that support the function of the brain. They perform many roles, such as providing nutrients to the neurons, maintaining the extracellular ion balance and participating in the blood brain barrier
Blood brain barrier (BBB): Interface separating the blood and brain tissue. This barrier is highly selective, allowing control of the composition of the ISF
Brain parenchyma: The functional part of the brain composed of neurons and glial cells
Cerebral cortex: The brain’s outer gray matter layer, consisting primarily of cell bodies, playing an important role *e.g.* in attention, awareness, thought, language, and consciousness, and initiation of motor functions
Cerebrospinal fluid (CSF): Fluid that bathes the brain and spinal cord. It is located in the ventricles and subarachnoid space of the brain
Choroid plexus: Tuft of highly vascular specialised epithelium located in the ventricles of the brain and responsible for producing CSF
Circle of Willis: The ring of arterial vessels located at the base of the brain and responsible for connecting the main arterial trunks. Acts as a safeguard to arterial supply to the brain in the event of a vascular occlusion
Contusion: A bruise caused when capillaries are damaged, allowing blood to seep into the tissue
Craniectomy: Neurosurgical procedure, in which a section of the skull is removed to help relieve increased intracranial pressure
Craniosynostosis: Birth defect in which one or more fibrous sutures of the infant skull fuse prematurely, changing the growth pattern of the skull
Cytotoxic edema: Swelling of individual cells. It is caused when cellular metabolism is disrupted, which prevents the cell from maintaining an osmotic equilibrium with the interstitial fluid, and allows water to accumulate in the cell
Diffuse axonal injury (DAI): Brain damage in the form of extensive lesions in nerve fibres in the white matter. Seen in a number of conditions including deprivation of oxygen or blood supply and one of the most devastating results of brain trauma
Donnan effect: Where immobile fixed charge density induces a greater osmotic pressure in the tissue than its surroundings
Edema: Swelling of soft tissue due to excess fluid accumulation
Fixed charge density (FCD): A (usually negative) charge on the solid components (e.g., extracellular and intracellular matrix) of biological tissue
Forament magnum: A large opening at the base of the skull tthrough which the spinal cord passes into the cranial cavity
Glial cells: Non-neuronal cells in the brain that support functions not directly related to electrical signal propagation
Glioblastoma multiforme: Highly malignant, fast growing type of brain cancer arising from astrocytes
Gyrification: Process and extent of folding of the cerebral cortex as a consequence of brain growth during embryonic and early postnatal development
Gyrification index: Ratio between total contour surface of the brain and its convex hull
Head injury criterion (HIC): Measure of likelihood of head injury caused by impact, used to assess safety of vehicles, protective gear, and sport equipment
Hydrocephalus: Abnormal accumulation of CSF in the ventricles (causing ventricle expansion and tissue deformation)
Infarction: Regional of tissue death, caused by lack of blood supply (ischaemia) and oxygen (hypoxia)
Interstitial fluid (ISF): Solution that bathes and surrounds brain cells
Ischemia: Inadequate blood supply to a local area due to blockage of blood vessels (leading to lack of oxygen)
Lissencephaly: Developmental malformation of the human brain caused by defective cortical development resulting in inadequate gyration and a partly or wholly smooth brain surface
Monro Kellie hypothesis: Constant sum of venous volume, arterial volume, brain volume, and cerebrospinal fluid volume
Morphogenesis: Biological process that causes an organism to develop its shape
Neurons: Electrically excitable cells that transmit information through the brain, via electrical and chemical signals
Polymicrogyria: Developmental malformation of the human brain characterized by focal replacement of the cortex by an excessive number of of small convolutions
Sagittal sinuses: One of the large venous channels (sinuses) found in the dura and responsible for draining all venous blood out of the brain and into the veins of the neck
Subarachnoid space (SAS): Space between the membrane layers on the outer surface of the brain, containing CSF
Traumatic brain injury (TBI): Intracranial injury caused by an external force that traumatically injures the brain, major cause of death and disability worldwide
Vasogenic edema: Brain swelling caused by a breakdown of the BBB
Ventricles: Communicating network of CSF-filled cavities, located within the brain

## References

[CR1] Abbot NJ (2004). Evidence for bulk flow of brain interstitial fluid: significance for physiology and pathology. Neurochem Int.

[CR2] Abbott NJ, Ronnback L, Hansson E (2006). Astrocyte-endothelial interactions at the blood-brain barrier. Nat Rev Neurosci.

[CR3] Alastruey J, Parker KH, Peiro J, Byrd SM, Sherwin SJ (2007). Modelling the circle of Willis to assess the effects of anatomical variations and occlusions on cerebral flows. J Biomech.

[CR4] Al-Bsharat AS, Hardy WN, Yang KH, Khalil TB, Tashman S, King AI (1999) Brain/skull relative displacement magnitude due to blunt head impact: new experimental data and model. Proceedings of 43rd Stapp Car Crash Conference, 101–160

[CR5] Alford PW, Dabiri BE, Goss JA, Hemphill MA, Brigham MD, Parker KK (2011). Blast-induced phenotypic switching in cerebral vasospasm. Proc Natl Acad Sci.

[CR6] Alnaes MS, Isaksen J, Mardal KA, Romner B, Morgan MK, Ingebrigtsen T (2007). Computation of hemodynamics in the circle of willis. Stroke.

[CR7] Ambrosi D, Ateshian GA, Arruda EM, Cowin SC, Dumais J, Goriely A, Holzapfel GA, Humphrey JD, Kemkemer R, Kuhl E, Olberding JE, Taber LA, Garikipati K (2011). Perspectives on biological growth and remodeling. J Mech Phys Solids.

[CR8] Ateshian GA, Likhitpanichkul M, Hung CT (2006). A mixture theory analysis for passive transport in osmotic loading of cells. J Biomech.

[CR9] Attolini CSO, Cheng Y-K, Beroukhim R, Getz G, Abdel-Wahab O, Levine RL, Mellinghoff IK, Michor F (2010). A mathematical framework to determine the temporal sequence of somatic genetic events in cancer. Proc Natl Acad Sci.

[CR10] Babbs CF, Shi R (2013). Subtle paranodal injury slows impulse conduction in a mathematical model of myelinated axons. PLoS ONE.

[CR11] Bain AC, Meaney DF (2000). Tissue-level thresholds for axonal damage in an experimental model of central nervous system white matter injury. J Biomech Eng.

[CR12] Bain AC, Raghupathi R, Meaney DF (2001). Dynamic stretch correlates to both morphological abnormalities and electrophysiological impairment in a model of traumatical axonal injury. J Neurotrauma.

[CR13] Basanta D, Simon M, Hatzikirou H, Deutsch A (2008). Evolutionary game theory elucidates the role of glycolysis in glioma progression and invasion. Cell Prolif.

[CR14] Bayly PV, Cohen TS, Leister EP, Ajo D, Leuthardt EC, Genin GM (2005). Deformation of the human brain induced by mild acceleration. J Neurotrauma.

[CR15] Bayly PV, Clayton EH, Genin GM (2012). Quantitative imaging methods for the development and validation of brain biomechanics models. Annu Rev Biomed Eng.

[CR16] Bayly PV, Okamoto R, Xu G, Shi Y, Taber LA (2013). A cortical folding model incorporating stress-dependent growth explains gyral wavelengths and stress patterns in the developing brain. Phys Biol.

[CR17] Bayly PV, Taber LA, Kroenke CD (2014). Mechanical forces in cerebral cortical folding: a review of measurements and models. J Mech Behav Biomed Mater.

[CR18] Ben Amar M, Goriely A (2005). Growth and instability in elastic tissues. J Mech Phys Solids.

[CR19] Bhardwaj A (2007). Osmotherapy in neurocritical care. Curr Neurol Neurosci Rep.

[CR20] Bhardwaj G, Chowdhury V, Jacobs MB, Moran KT, Martin FJ, Coroneo MT (2010). A systematic review of the diagnostic accuracy of ocular signs in pediatric abusive head trauma. Ophthalmology.

[CR21] Biot MA (1957). Folding instability of a layered viscoelastic medium under compression. Proc R Soc Lond A.

[CR22] Bilston LE, Liu Z, Phan-Thien N (2001). Large strain behavior of brain tissue in shear: some experimental data and differential constitutive model. Biorheology.

[CR23] Bowen RM (1967). Toward a thermodynamics and mechanics of mixtures. Arch Ration Mech Anal.

[CR24] Boucher PA, Joós B, Morris CE (2012). Coupled left-shift of Nav channels: modeling the Na+-loading and dysfunctional excitability of damaged axons. J Comput Neurosci.

[CR25] Bray D (1984). Axonal growth in response to experimentally applied mechanical tension. Dev Biol.

[CR26] Brooks D, Gabella B, Hoffman R, Sosin D, Whiteneck G (1997). Traumatic brain injury: designing and implementing a population-based follow-up system. Arch Phys Med Rehabil.

[CR27] Budday S, Raybaud C, Kuhl E (2014). A mechanical model predicts morphological abnormalities in the developing human brain. Sci Rep.

[CR28] Budday S, Steinmann P, Kuhl E (2014). The role of mechanics during brain development. J Mech Phys Solids.

[CR29] Budday S, Kuhl E, Hutchinson JW (2015a) Period-doubling and period-tripling in growing bilayered systems. Phil Mag doi:10.1080/14786435.2015.1014443 10.1080/14786435.2015.1014443 PMC4704805 26752977

[CR30] Budday S, Nay R, de Rooij R, Steinmann P, Wyrobek T, Ovaert TC, Kuhl E (2015b) Mechanical properties of gray and white matter brain tissue by indentation. J Mech Behav Biomed Mater (in press) 10.1016/j.jmbbm.2015.02.024 PMC4395547 25819199

[CR31] Budday S, Steinmann P, Goriely A, Kuhl E (2015c) Morphogenesis and pattern formation of the mammalian brain (submitted)

[CR32] Caffey J (1972). On the theory and practice of shaking infants. Its potential residual effects of permanent brain damage and mental retardation. Am J Dis Child.

[CR33] Calder IM, Hill I, Scholtz CL (1984). Primary brain trauma in non-accidental injury. J Clin Pathol.

[CR34] Cassot F, Zagzoule M, Marc-Vergnes JP (2000). Hemodynamic role of the circle of Willis in stenoses of internal carotid arteries: an analytical solution of a linear model. J Eng Math.

[CR35] Cebral JR, Castro MA, Soto O, Lohner R, Alperin N (2003). Blood-flow models of the circle of Willis from magnetic resonance data. J Biomech.

[CR36] Cernak I, Noble-Haeusslein LJ (2010). Traumatic brain injury: an overview of pathobiology with emphasis on military populations. J Cereb Blood Flow Metab.

[CR37] Chatelin S, Vappou J, Roth S, Raul JS, Willinger R (2012). Towards child versus adult brain mechanical properties. J Mech Behav Biomed Mater.

[CR38] Chen Y, Ostoja-Starzewski M (2010). MRI-based finite element modeling of head trauma: spherically focusing shear waves. Acta Mech.

[CR39] Chen J, Wang XM, Luan LM, Chao BT, Pang B, Song H, Pang Q (2012). Biological characteristics of the cerebral venous system and its hemodynamic response to intracranial hypertension. Chin Med J.

[CR40] Christian CW, Block R (2009). Abusive head trauma in infants and children. Pediatrics.

[CR41] Chung RS, Staal JA, McCormack GH, Dickson TC, Cozens MA, Chuckowree JA, Quilty MC, Vickers JC (2005). Mild axonal stretch injury in vitro induces a progressive series of neurofilament alterations ultimately leading to delayed axotomy. J Neurotrauma.

[CR42] Clarke MJ, Meyer FB (2007). The history of mathematical modeling in hydrocephalus. Neurosurg Focus.

[CR43] Cloots RJH, Gervaise HMT, Van Dommelen JAW, Geers MGD (2008). Biomechanics of traumatic brain injury: influences of the morphologic heterogeneities of the cerebral cortex. Ann Biomed Eng.

[CR44] Cloots RJH, Van Dommelen JAW, Nyberg T, Kleiven S, Geers MGD (2011). Micromechanics of diffuse axonal injury: influence of axonal orientation and anisotropy. Biomech Model Mechanbiol.

[CR45] Cloots RJH, Van Dommelen JAW, Geers MGD (2012). A tissue-level anisotropic criterion for brain injury based on microstructural axonal deformation. J Mech Behav Biomed.

[CR46] Cloots RJH, van Dommelen JAW, Kleiven S, Geers MGD (2013). Multi-scale mechanics of traumatic brain injury: predicting axonal strains from head loads. Biomech Model Mechanobiol.

[CR47] Connell S, Gao J, Chen J, Shi R (2011) Novel model to investigate blast injury in the central nervous system. J Neurotrauma 28:1229–1236 10.1089/neu.2011.1832 21529318

[CR48] Cooper DJ, Rosenfeld JV, Murray L, Arabi YM, Davies AR, D’Urso P, Kossmann T, Ponsford J, Seppelt I, Reilly P, Wolfe R (2011). Decompressive craniectomy in diffuse traumatic brain injury. N Engl J Med.

[CR49] David T, Moore S (2008). Modelling perfusion in the cerebral vasculature. Med Eng Phys.

[CR50] Deck C, Willinger R (2008). Improved head injury criteria based on head FE model. Int J Crashworthiness.

[CR51] Donnan FG (1924) The theory of membrane equilibria. Chem Rev 1:73–90

[CR52] Donnelly BR, Medige J (1997). Shear properties of human brain tissue. J Biomech Eng.

[CR53] Dorfmann A, Ogden RW (2004). A constitutive model for the mullins effect with permanent set in particle-reinforced rubber. Int J Solids Struct.

[CR54] Drapaca CS, Tenti G, Rohlf K, Sivaloganathan S (2006). A quasi-linear viscoelastic constitutive equation for the brain: applications to hydrocephalus. J Elast.

[CR55] Drapaca CS, Fritz JS (2012). A mechano-electrochemical model of brain neuro-mechanics: application to normal pressure hydrocephalus. Int J Numer Anal Model Ser B.

[CR56] Dronne MA, Boissel JP, Grenier E (2006). A mathematical model of ion movements in grey matter during a stroke. J Theor Biol.

[CR57] Duhaime AC, Gennarelli TA, Thibault LE, Bruce DA, Margulies SS, Wiser R (1987). The shaken baby syndrome. A clinical, pathological, and biomechanical study. J Neurosurg.

[CR58] Duhaime AC, Christian CW, Rorke LB, Zimmerman RA (1998). Nonaccidental head injury in infants—the “Shaken-Baby Syndrome”. N Engl J Med.

[CR59] Duncan CC, Summers AC, Perla EJ, Coburn KL, Mirsky AF (2011). Evaluation of traumatic brain injury: brain potentials in diagnosis, function, and prognosis. Int J Psychophysiol.

[CR60] Ehlers W, Wagner A (2015). Multi-component modelling of human brain tissue: a contribution to the constitutive and computational description of deformation, flow and diffusion processes with application to the invasive drug-delivery problem. Comput Methods Biomech Biomed Eng.

[CR61] Eley KA, Johnson D, Wilkie AO, Jayamohan J, Richards P, Wall SA (2012). Raised intracranial pressure is frequent in untreated nonsyndromic unicoronal synostosis and does not correlate with severity of phenotypic features. Plast Reconstr Surg.

[CR62] Elkin BS, Shaik MA, Morrison B (2010). Fixed negative charge and the Donnan effect: a description of the driving forces associated with brain tissue swelling and oedema. Philos Trans R Soc A.

[CR63] Elkin BS, Ilankova A, Morrison B (2011). Dynamic, regional mechanical properties of the porcine brain: indentation in the coronal plane. J Biomech Eng ASME.

[CR64] Elliott NSJ, Bertram CD, Martin BA, Brodbelt AR (2013). Syring-omyelia: a review of the biomechanics. J Fluids Struct.

[CR65] Elliott NSJ, Lockerby DA, Brodbelt AR (2011). A lumped-parameter model of the cerebrospinal system for investigating arterial-driven flow in post traumatic syringomyelia. Med Eng Phys.

[CR66] Emerson MV, Jakobs E, Green WR (2007). Ocular autopsy and histopathologic features of child abuse. Ophthalmology.

[CR67] Estes MS, McElhaney JH (1970). Response of brain tissue of compressive loading. ASME.

[CR68] Fallenstein GT, Hulce VD, Melvin JW (1969). Dynamic mechanical properties of human brain tissue. J Biomech.

[CR69] Feng Y, Abney TM, Okamoto RJ, Pless RB, Genin GM, Bayly PV (2010). Relative brain displacement and deformation during constrained mild frontal head impact. J Roy Soc Interface.

[CR70] Feng Y, Okamoto RJ, Namani R, Genin GM, Bayly PV (2013). Measurements of mechanical anisotropy in brain tissue and implications for transversely isotropic material models of white matter. J Mech Behav Biomed Mater.

[CR71] Ferrandez A, David T, Bamford J, Scott J, Guthrie A (2001). Computational models of blood flow in the circle of Willis. Comput Methods Biomech Biomed Eng.

[CR72] Fink ME (2012). Osmotherapy for intracranial hypertension: mannitol versus hypertonic saline. Contin Lifelong Learn Neurol Crit Care Neurol.

[CR73] Fletcher TL, Kolias AG, Hutchinson PJA, Sutcliffe MPF (2014). Development of a finite element model of decompressive craniectomy. PLoS ONE.

[CR74] Franceschini G, Bigoni D, Regitnig P, Holzapfel GA (2006). Brain tissue deforms similarly to filled elastomers and follows consolidation theory. J Mech Phys Solids.

[CR75] Galford JE, McElhaney JH (1969). Some viscoelastic properties of scalp, brain and dura. ASME 69-BHF.

[CR76] Gao CP, Ang BT (2008) Biomechanical modeling of decompressive craniectomy in traumatic brain injury. Acta Neurochir Suppl102:279–282 10.1007/978-3-211-85578-2_52 19388329

[CR77] Garcia JJ, Smith JH (2009). A biphasic hyperelastic model for the analysis of fluid and mass transport in brain tissue. Ann Biomed Eng.

[CR78] García-Grajales JA, Rucabado G, García-Dopico A, Peña JM, Jérusalem A (2014) Neurite, a finite difference large scale parallel program for the simulation of electrical signal propagation in neurites under mechanical loading. PLoS One 10(2):e0116532 10.1371/journal.pone.0116532 PMC4334526 25680098

[CR79] Geddes JF, Vowles GH, Hackshaw AK, Nickols CD, Scott IS, Whitwell HL (2001). Neuropathology of inflicted head injury in children. II. Microscopic brain injury in infants. Brain.

[CR80] Gefen A, Margulies SS (2004). Are in vivo and in situ brain tissues mechanically similar?. J Biomech.

[CR81] Gennarelli T (1993) Mechanisms of brain injury. J Emerg Med 11:5–11 8445204

[CR82] Gilles EE, McGregor ML, Levy-Clarke G (2003). Retinal hemorrhage asymmetry in inflicted head injury: a clue to pathogenesis?. J Pediatr.

[CR83] Gilles F, Nelson M (2012). The developing human brain: growth and adversities.

[CR84] Giordano C, Cloots RJH, Van Dommelen JAW, Kleiven S (2014). The influence of anisotropy in a computational head model for brain injury prediction. J Biomech.

[CR85] Goldsmith W (2001). The state of head injury biomechanics: past, present, and future: part 1. Crit Rev Biomed Eng.

[CR86] Goldstein LE, Fisher AM, Tagge CA, Zhang XL, Velisek L, Sullivan JA, Upreti C, Kracht JM, Ericsson M, Wojnarowicz MW, Goletiani CJ, Maglakelidze GM, Casey N, Moncaster JA, Minaeva O, Moir RD, Nowinski CJ, Stern RA, Cantu RC, Geiling J, Blusztajn JK, Wolozin BL, Ikezu T, Stein TD, Budson AE, Kowall NW, Chargin D, Sharon A, Saman S, Hall GF, Moss WC, Cleveland RO, Tanzi E, Stanton PK, McKee AC (2012). Chronic traumatic encephalopathy in blast-exposed military veterans and a blast neurotrauma mouse model. Sci Transl Med.

[CR87] Graham DI, Raghupathi R, Saatman KE, Meaney D, McIntosh TK (2000). Tissue tears in the white matter after lateral fluid percussion brain injury in the rat: relevance to human brain injury. Acta Neuropathol.

[CR88] Grände P, Romner B (2012). Osmotherapy in brain edema: a questionable therapy. J Neurosurg Anest.

[CR89] Green MA, Bilston LE, Sinkus R (2008). In vivo brain viscoelastic properties measured by magnetic resonance elastography. NMR Biomed.

[CR90] Gupta RK, Przekwas A (2013) Mathematical models of blast-induced TBI: current status, challenges, and prospects. Front Neurol 4:1–21 10.3389/fneur.2013.00059 PMC3667273 23755039

[CR91] Guthkelch AN (1971). Infantile subdural haematoma and its relationship to whiplash injuries. Br Med J.

[CR92] Haines DE, Harkey HL, al Mefty O (1993). The “subdural” space: a new look at an outdated concept. Neurosurgery.

[CR93] Hakim S, Jimenez A, Rosas F (1955). Drainage of the cerebrospinal fluid into the spinal epidural space: a new technique for the treatment of hydrocephalus. Acta Neurochir.

[CR94] Hamhaber U, Klatt D, Papazoglou S, Hollmann M, Stadler J, Sack I, Bernarding J, Braun J (2010). In vivo magnetic resonance elastography of human brain at 7 T and 1.5 T. J Magn Reson Imaging.

[CR95] Harding B, Risdon RA, Krous HF (2004). Shaken baby syndrome. Br Med J.

[CR96] Hardy WN, Foster CD, Mason MJ, Yang KH, King AI, Tashman S (2001). Investigation of head injury mechanisms using neutral density technology and high-speed biplanar X-ray. Stapp Car Crash J.

[CR97] Haslach HW, Leahy LN, Riley P, Gullapalli R, Xu S, Hsieh AH (2014). Solid-extracellular fluid interaction and damage in the mechanical response of rat brain tissue under confined compression. J Mech Behav Biomed Mater.

[CR98] Hatten ME (1999). Central nervous system neuronal migration. Annu Rev Neurosci.

[CR99] Hicks RR, Fertig SJ, Desrocher RE, Koroshetz WJ, Pancrazio JJ (2010). Neurological effects of blast injury. J Trauma Inj Infect Crit Care.

[CR100] Ho J, Kleiven S (2009). Can sulci protect the brain from traumatic injury?. J Biomech.

[CR101] Hodgkin AL, Huxley AF (1952). A quantitative description of membrane current and its application to conduction and excitation in nerve. J Physiol.

[CR102] Holland MA, Miller KE, Kuhl E (2014) Emerging brain morphologies from axonal elongation (submitted) 10.1007/s10439-015-1312-9 PMC4497873 25824370

[CR103] Holzapfel GA, Ogden RW (2009). On planar biaxial tests for anisotropic nonlinearly elastic solids. A continuum mechanical framework. Math Mech Solids.

[CR104] Hrapko M, Van Dommelen JAW, Peters GWM, Wismans JSHM (2006). The mechanical behaviour of brain tissue: large strain response and constitutive modelling. Biorheology.

[CR105] Hrapko M, Van Dommelen JAW, Peters GWM, Wismans JSHM (2008). The influence of test conditions on characterisation of the mechanical properties of brain tissue. J Biomech Eng ASME.

[CR106] Hrapko M, Van Dommelen JAW, Peters GWM, Wismans JSHM (2009). On the consequences of non linear constitutive modelling of brain tissue for injury prediction with numerical head models. Int J Crashworthiness.

[CR107] Hue CD, Cao S, Haider SF, Vo KV, Effgen GB, Vogel E, Panzer MB, Bass CRD, Meaney DF, Morrison B (2013). Blood-brain barrier dysfunction after primary blast injury in vitro. J Neurotrauma.

[CR108] Huyghe JM, Janssen JD (1997). Quadriphasic mechanics of swelling incompressible porous media. Int J Eng Sci.

[CR109] Hymel KP, Stoiko MA, Herman BE, Combs A, Harper NS, Lowen D, Deye KP, Homa K, Blackman JA (2010). Head injury depth as an indicator of causes and mechanisms. Pediatrics.

[CR110] Ibrahim NG, Margulies SS (2010). Biomechanics of the toddler head during low-height falls: an anthropomorphic dummy analysis. J Neurosurg Pediatr.

[CR111] Ibrahim NG, Natesh R, Szczesny SE, Ryall K, Eucker SA, Coats B, Margulies SS (2010). In situ deformations in the immature brain during rapid rotations. J Biomech Eng.

[CR112] Ivarsson J, Viano DC, Lövsund P, Aldman B (2000). Strain relief from the cerebral ventricles during head impact: experimental studies on natural protection of the brain. J Biomech.

[CR113] Jaspan T, Narborough G, Punt JA, Lowe J (1992). Cerebral contusional tears as a marker of child abuse-detection by cranial sonography. Pediatr Radiol.

[CR114] Jean A, Nyein MK, Zheng JQ, Moore DF, Joannopoulos JD, Radovitzky RA (2014) An animal-to-human scaling law for blast-induced traumatic brain injury risk assessment. Proc Natl Acad Sci. doi:10.1073/pnas.1415743111 10.1073/pnas.1415743111 PMC4217421 25267617

[CR115] Jeevan DS, Anlsow P, Jayamohan J (2008). Abnormal venous drainage in syndromic craniosynostosis and the role of CT venography. Childs Nerv Syst.

[CR116] Jennett B (1996). Epidemiology of head injury. J Neurol Neurosurg Psychiatry.

[CR117] Jérusalem A, Dao M (2012). Continuum modeling of neuronal cell under blast loading. Acta Biomater.

[CR118] Jérusalem A, García-Grajales JA, Merchán-Perez A, Peña JM (2014) A computational model coupling mechanics and electrophysiology in spinal cord injury. Biomech Model Mechanobiol 13:883–896 10.1007/s10237-013-0543-7 24337934

[CR119] Jin X, Zhu F, Mao H, Shen M, Yang KH (2013). A comprehensive experimental study on material properties of human brain tissue. J Biomech.

[CR120] Johnson CL, McGarry MDJ, Gharibans AA, Weaver JB, Paulsen KD, Wang H, Olivero WC, Sutton BP, Georgiadis JG (2013). Local mechanical properties of white matter structures in the human brain. Neuroimage.

[CR121] Jung A, Falterneier R, Rothoerl R, Brawanski A (2005). A mathematical model of cerebral circulation and oxygen supply. Math Biol.

[CR122] Kansal AR, Torquato S, Harsh GR, Chiocca EA, Deisboeck TS (2000). Simulated brain tumor growth dynamics using a three-dimensional cellular automaton. J Theor Biol.

[CR123] Kawamata T, Mori T, Sato S, Katayama Y (2007) Tissue hyperosmolality and brain edema in cerebral contusion. Neurosurg Focus 22:1–8 10.3171/foc.2007.22.5.6 17613236

[CR124] Kellie G (1824). Appearances observed in the dissection of two individuals; death from cold and congestion of the brain. Trans Med Chir Soc Edinb.

[CR125] Kharatishvili I, Sierra A, Immonen RJ, Gröhn OHJ, Pitkänen A (2009). Quantitative T2 mapping as a potential marker for the initial assessment of the severity of damage after traumatic brain injury in rat. Exp Neurol.

[CR126] Kleiven S (2007). Predictors for traumatic brain injuries evaluated through accident reconstruction. Stapp Car Crash J.

[CR127] Knutsen AK, Chang YV, Grimm CM, Phan L, Taber LA, Bayly PV (2010). A new method to measure cortical growth in the developing brain. J Biomech Eng.

[CR128] Knutsen AK, Kroenke CD, Chang YV, Taber LA, Bayly PV (2013). Spatial and temporal variations of cortical growth during gyrogenesis in the developing ferret brain. Cereb Cortex.

[CR129] Koch C (1999). Biophysics of computation.

[CR130] Kruse SA, Rose GH, Glaser KJ, Manduca A, Felmlee JP, Rack CR, Ehman RL (2008). Magnetic resonance elastography of the brain. Neuroimage.

[CR131] Kronik N, Kogan Y, Vainstein V, Agur Z (2008). Improving alloreactive CTL immunotherapy for malignant gliomas using a simulation model of their interactive dynamics. Cancer Immunol Immunother.

[CR132] Kurtcuoglu V, Poulikakos D, Ventikos Y (2005). Computational modeling of the mechanical behaviour of the cerebrospinal fluid system. ASME J Biomech Eng.

[CR133] Kurtcuoglu V, Miller K (2011). Computational fluid dynamics and its assessment of cerebrospinal fluid flow and its coupling with cerebral blood flow. Biomechanics of the brain.

[CR134] Kyriacou SK, Mohamed A, Miller K, Neff S (2002). Brain mechanics for neurosurgery: modeling issues. Biomech Model Mechanobiol.

[CR135] Lai WM, Hou JS, Mow VC (1991). A triphasic theory for the swelling and deformation behavior of articular cartilage. J Biomech Eng.

[CR136] Lang GE (2014) Mechanics of swelling and damage in brain tissue: a theoretical approach DPhil Thesis, University of Oxford

[CR137] Lang G, Stewart PS, Vella D, Waters SL, Goriely A (2014). Is the Donnan effect sufficient to explain swelling in brain tissue slices?. J R Soc Interface.

[CR138] Lang G, Vella D, Waters SL, Goriely A (2015). Propagation of damage in brain tissue: coupling the mechanics of edema and oxygen delivery. submitted for publication 10.1007/s10237-015-0665-1 25822263

[CR139] Lauret C, Hrapko M, Van Dommelen JAW, Peters GWM, Wismans JSHM (2009). Optical characterization of acceleration-induced strain fields in inhomogeneous brain slices. Med Eng Phys.

[CR140] Lauritzen M, Dreier JP, Fabricius M, Hartings JA, Graf R, Strong AJ (2011). Clinical relevance of cortical spreading depression in neurological disorders: migraine, malignant stroke, subarachnoid and intracranial hemorrhage, and traumatic brain injury. J Cereb Blood Flow Metab.

[CR141] Leder K, Pitter K, LaPlant Q, Hambardzumyan D, Ross BD, Chan TA, Holland EC, Michor F (2014). Mathematical modeling of PDGF-driven glioblastoma reveals optimized radiation dosing schedules. Cell.

[CR142] Liang D, Bhatta S, Gerzanich V, Simard JM (2007). Cytotoxic edema: mechanisms of pathological cell swelling. Neurosurg Focus.

[CR143] Leung LY, VandeVord PJ, Dal Cengio AL, Bir C, Yang KH, King AI (2008). Blast related neurotrauma: a review of cellular injury. Mol Cell Biomech.

[CR144] Levine D (1999). The pathogenesis of normal pressure hydrocephalus: a theoretical analysis. Bull Math Biol.

[CR145] Lindenberg R, Freytag E (1969). Morphology of brain lesions from blunt trauma in early infancy. Arch Pathol.

[CR146] Linninger AA, Somayaji MR, Erickson T, Guo X, Penn RD (2008). Computational methods for predicting drug transport in anisotropic and heterogeneous brain tissue. J Biomech.

[CR147] Linninger AA, Xenos M, Sweetman B, Ponkshe S, Guo X, Penn R (2009). A mathematical model of blood, cerebrospinal fluid and brain dynamics. Math Biol.

[CR148] Lundberg N, Troupp H, Lorin H (1965). Continuous recording of the ventricular-fluid pressure in patients with severe acute traumatic brain injury. A preliminary report. J Neurosurg.

[CR149] Mack J, Squier W, Eastman JT (2009). Anatomy and development of the meninges: implications for subdural collections and CSF circulation. Pediatr Radiol.

[CR150] Margulies SS, Thibault LE, Gennarelli TA (1998). Physical model simulations of brain injury in the primate. J Biomech.

[CR151] Marucci DD, Johnston CP, Anslow P, Jayamohan J, Richards PG, Wilkie AO, Wall SA (2008). Implications of a vertex bulge following modified strip craniectomy for sagittal synostosis. Plast Reconstr Surg.

[CR152] Matshes E (2010). Retinal and optic nerve sheath haemorrhages are not pathognomonic of abusive head injury.

[CR153] Maxwell WL, Domleo A, McColl G, Jafari SS, Graham DI (2003). Post-acute alterations in the axonal cytoskeleton after traumatic axonal injury. J Neurotrauma.

[CR154] Meaney DF, Morrison B, Bass CD (2014) The mechanics of traumatic brain injury: a review of what we know and what we need to know for reducing its societal burden. J Biomech Eng ASME 136:021008 10.1115/1.4026364 PMC4023660 24384610

[CR155] Mehrabian A, Abousleiman Y (2011). General solutions to poroviscoelastic model of hydrocephalic human brain tissue. J Theor Biol.

[CR156] Menzel A, Kuhl E (2012). Frontiers in growth and remodeling. Mech Res Commun.

[CR157] Miller K, Chinzei K (1997). Constitutive modelling of brain tissue: experiment and theory. J Biomech.

[CR158] Miller K, Chinzei K (2002). Mechanical properties of brain tissue in tension. J Biomech.

[CR159] Miller K, Chinzei K, Orssengo G, Bednarz P (2000). Mechanical properties of brain tissue in vivo: experiment and computer simulation. J Biomech.

[CR160] Mokri B (2001). The Monro–Kellie hypothesis: applications in CSF volume depletion. Neurology.

[CR161] Monro A (1783) Observations on structure and functions of the nervous system. Creech and Johnson, Edinburgh

[CR162] Moore DF, Jérusalem A, Nyein M, Noels L, Jaffee MS, Radovitzky RA (2009). Computational biology—modeling of primary blast effects on the central nervous system. NeuroImage.

[CR163] Morrison B, Saatman K, Meaney D, McIntosh T (1998). In vitro central nervous system models of mechanically induced trauma: a review. J Neurotrauma.

[CR164] Morrison B, Cater HL, Benham CD, Sundstrom LE (2006). An in vitro model of traumatic brain injury utilising two-dimensional stretch of organotypic hippocampal slice cultures. J Neurosci Methods.

[CR165] Morritt DG, Yeh FJ, Wall SA, Richards PG, Jayamohan J, Johnson D (2010). Management of isolated sagittal synostosis in the absence of scaphocephaly: a series of eight cases. Plast Reconstr Surg.

[CR166] Nahum AM, Smith R, Ward CC (1977) Intracranial pressure dynamics during head impact. Proceedings of 21th Stapp Car Crash Conference, 339–366

[CR167] NHTSA (1972) Occupant crash protection—head injury criterion, S6.2 of FMVSS 571.208. NHTSA, Washington

[CR168] Nicholson C (2001). Diffusion and related transport mechanisms in brain tissue. Rep Prog Phys.

[CR169] Nyein M, Jason AM, Yu L, Pita CM, Joannopoulos JD, Moore DF, Radovitzky RA (2010). In silico investigation of intracranial blast mitigation with relevance to military traumatic brain injury. Proc Natl Acad Sci.

[CR170] Oehmichen M, Schleiss D, Pedal I, Saternus KS, Gerling I, Meissner C (2008). Shaken baby syndrome: re-examination of diffuse axonal injury as cause of death. Acta Neuropathol.

[CR171] Oreskovic D, Klarica M (2010). The formation of cerebrospinal fluid: nearly a hundred years of interpretations and misinterpretations. Brain Res Rev.

[CR172] Ouyang H, Galle B, Li J, Nauman E, Shi R (2008). Biomechanics of spinal cord injury: a multimodal investigation using ex vivo guinea pig spinal cord white matter. J Neurotrauma.

[CR173] Ouyang H, Sun W, Fu Y, Li J, Cheng J, Nauman E, Shi R (2010). Compression induces acute demyelination and potassium channel exposure in spinal cord. J Neurotrauma.

[CR174] Papadopoulos M, Saadoun S, Binder D, Manley G, Krishna S, Verkman A (2004). Molecular mechanisms of brain tumor edema. Neuroscience.

[CR175] Papastavrou A, Steinmann P, Kuhl E (2013). On the mechanics of continua with boundary energies and growing surfaces. J Mech Phys Solids.

[CR176] Paulson OB, Strandgaard R, Edvinsson L (1990). Cerebral autoregulation. Cerebrovasc Brain Metab Rev.

[CR177] Peter SJ, Mofrad MRK (2012). Computational modeling of axonal microtubule bundles under tension. Biophys J.

[CR178] Pfrieger FW (2010). Role of glial cells in the formation and maintenance of synapses. Brain Res Rev.

[CR179] Powathil G, Kohandel M, Sivaloganathan S, Oza A, Milosevic M (2007). Mathematical modeling of brain tumors: effects of radiotherapy and chemotherapy. Phys Med Biol.

[CR180] Prange MT, Margulies SS (2002). Regional, directional, and age-dependent properties of the brain undergoing large deformation. J Biomech Eng.

[CR181] Prevost TP, Balakrishnan A, Suresh S, Socrate S (2011). Biomechanics of brain tissue. Acta Biomater.

[CR182] Raslan A, Bhardwaj A (2007). Medical management of cerebral edema. Neurosurg Focus.

[CR183] Rashid B, Destrade M, Gilchrist MD (2012). Mechanical characterization of brain tissue in compression at dynamic strain rates. J Mech Behav Biomed Mater.

[CR184] Rashid B, Destrade M, Gilchrist MD (2013). Mechanical characterization of brain tissue in simple shear at dynamic strain rates. J Mech Behav Biomed Mater.

[CR185] Rashid B, Destrade M, Gilchrist MD (2014). Mechanical characterization of brain tissue in tension at dynamic strain rates. J Mech Behav Biomed Mater.

[CR186] Raybaud C, Widjaja E (2011). Development and dysgenesis of the cerebral cortex: malformations of cortical development. Neuroimaging Clin N Am.

[CR187] Raybaud C, Ahmad T, Rastegar N, Shroff M, Al Nassar M (2013). The premature brain: developmental and lesional anatomy. Neuroradiology.

[CR188] Redzic ZB, Preston JE, Duncan JA, Chodobski A, Szmydynger-Chodobska J (2005) The choroid plexus-cerebrospinal fluid system: from development to aging. Curr Top Dev Biol 71:1–52 10.1016/S0070-2153(05)71001-2 16344101

[CR189] Reichard RR, White CL, Hladik CL, Dolinak D (2003). Beta-amyloid precursor protein staining of nonaccidental central nervous system injury in pediatric autopsies. J Neurotrauma.

[CR190] Richman DP, Stewart RM, Hutchinson JW, Caviness VS (1975). Mechanical model of brain convolutional development. Science.

[CR191] Risberg J, Lundberg N, Ingvar DH (1969). Regional cerebral blood volume during acute transient rises of the intracranial pressure (plateau waves). J Neurosurg.

[CR192] Rockne R, Alvord EC, Rockhill JK, Swanson KR (2009). A mathematical model for brain tumor response to radiation therapy. J Math Biol.

[CR193] Romano A, Scheel M, Hirsch S, Braun J, Sack I (2012). In vivo waveguide elastography of white matter tracts in the human brain. Magn Reson Med.

[CR194] Rosso L, Brock CS, Gallo JM, Saleem A, Price PM, Turkheimer FE, Aboagye EO (2009). A new model for prediction of drug distribution in tumor and normal tissues: pharmacokinetics of temozolomide in glioma patients. Cancer Res.

[CR195] Roth G, Dicke U (2005). Evolution of the brain and intelligence. Trends Cogn Sci.

[CR196] Sabet AA, Christoforou E, Zatlin B, Genin GM, Bayly PV (2008). Deformation of the human brain induced by mild angular head acceleration. J Biomech.

[CR197] Sack I, Beierbach B, Hamhaber U, Klatt D, Braun J (2008). Non-invasive measurement of brain viscoelasticity using magnetic resonance elastography. NMR Biomed.

[CR198] Sack I, Streitberger KJ, Krefting D, Paul F, Braun J (2011). The influence of physiological aging and atrophy on brain viscoelastic properties in humans. PLoS One.

[CR199] Schrot RJ, Muizelaar JP (2002). Mannitol in acute traumatic brain injury. Lancet.

[CR200] Shahim K, Drezet JM, Martin BA, Momjian S (2012). Ventricle equilibrium position in healthy and normal pressure hydrocephalus brains using an analytical model. ASME J Biomech Eng.

[CR201] Sharan M, Popel AS (2002). A compartment model for oxygen transport in brain microcirculation in the presence of blood substitutes. J Theor Biol.

[CR202] Shepherd GM (2004). The synaptic organization of the brain.

[CR203] Shi R, Whitebone J (2006). Conduction deficits and membrane disruption of spinal cord axons as a function of magnitude and rate of strain. J Neurophys.

[CR204] Siyahhan B, Knobloch V, de Zelicourt D, Asgari M, Schmid Daners M, Poulikakos D, Kurtcuoglu V (2014). Flow induced by ependymal cilia dominates near-wall cerebrospinal fluid dynamics in the lateral ventricles. J R Soc Interface.

[CR205] Sforza DM, Putman CM, Cebral JR (2009). Hemodynamics of cerebral aneurysms. Ann Rev Fluid Mech.

[CR206] Simard J, Kent T, Chen M, Tarasov KV, Gerzanich V (2007). Brain oedema in focal ischaemia: molecular pathophysiology and theoretical implications. Lancet Neurol.

[CR207] Soustiel JF, Sviri GE, Mahamid E, Shik V, Abeshaus S, Zaaroor M (2010). Cerebral blood flow and metabolism following decompressive craniectomy for control of increased intracranial pressure. Neurosurgery.

[CR208] Smillie A, Sobey I, Molnar Z (2005). A hydroelastic model of hydrocephalus. J Fluid Mech.

[CR209] Smith DH, Wolf JA, Lusardi RA, Lee VMY, Meaney DF (1999). High tolerance and delayed elastic response of cultured axons to dynamic stretch injury. J Neurosci.

[CR210] Smith DH, Meaney DF, Shull WH (2003). Diffuse axonal injury in head trauma. J Head Trauma Rehabil.

[CR211] Spaethling J, Meaney DF (2012). NMDA receptor mediated phosphorylation of GluR1 subunits contributes to the appearance of calcium-permeable AMPA receptors after mechanical stretch injury. Neurobiol Dis.

[CR212] Squier W, Lindberg E, Mack J, Darby S (2009). Demonstration of fluid channels in human dura and their relationship to age and intradural bleeding. Childs Nerv Syst.

[CR213] Squier W (2011). The “Shaken Baby” syndrome: pathology and mechanisms. Acta Neuropathol.

[CR214] Squier W, Jansen A (2014). Polymicrogyria: pathology, fetal origins and mechanisms. Acta Neuropathol Commun.

[CR215] Squier W, Mack J, Green A, Aziz T (2012). The pathophysiology of brain swelling associated with subdural hemorrhage: the role of the trigeminovascular system. Childs Nerv Syst.

[CR216] Stoverud KH, Darcis M, Helmig R, Majid Hassanizadeh S (2012). Modelling concentration distribution and deformation during convection-enhanced drug delivery into brain tissue. Transp Porous Med.

[CR217] Streitberger KJ, Sack I, Krefting D, Pfüller C, Braun J, Paul F, Wuerfel J (2012). Brain viscoelasticity alteration in chronic-progressive multiple sclerosis. PLoS ONE.

[CR218] Sun T, Hevner RF (2014). Growth and folding of the mammalian cerebral cortex: from molecules to malformations. Nat Rev Neurosci.

[CR219] Suresh S (2007). Biomechanics and biophysics of cancer cells. Acta Biomater.

[CR220] Swanson KR, Bridge C, Murray JD, Alvord EC (2003). Virtual and real brain tumors: using mathematical modeling to quantify glioma growth and invasion. J Neurol Sci.

[CR221] Sweetman B, Xenos M, Zitella L, Linninger AA (2011). Three-dimensional computational prediction of cerebrospinal fluid flow in the human brain. Comput Biol Med.

[CR222] Syková E, Nicholson C (2008). Diffusion in brain extracellular space. Physiol Rev.

[CR223] Terzaghi K (1943). Theoretical soil mechanics.

[CR224] Tracqui P, Cruywagen GC, Woodward DE, Bartoo GT, Murray JD, Alvord EC (1995). A mathematical model of glioma growth: the effect of chemotherapy on spatio-temporal growth. Cell Prolif.

[CR225] Tubbs RS, Loukas M, Louis RG, Shoja MM, Acakpo-Satchivi L, Blount JP, Salter EG, Oakes WJ, Wellons JC (2007). Anatomy of the falcine venous plexus. J Neurosurg.

[CR226] Tully B, Ventikos Y (2011). Cerebral water transport using multiple-network poroelastic theory: application to normal pressure hydrocephalus. J Fluid Mech.

[CR227] Tyler WJ (2012). The mechanobiology of brain function. Nat Rev Neurosci.

[CR228] Unterberg A, Stover J, Kress B, Kiening K (2004). Edema and brain trauma. Neuroscience.

[CR229] Ursino M, Lodi CA (1997). A simple mathematical model of the interaction between intracranial pressure and cerebral hemodynamics. J Appl Physiol.

[CR230] Van Dommelen JAW, Van der Sande TPJ, Hrapko M, Peters GWM (2010). Mechanical properties of brain tissue by indentation: interregional variation. J Mech Behav Biomed Mater.

[CR231] Van Dommelen JAW, Hrapko M, Peters GWM (2011) Constitutive modelling of brain tissue for prediction of traumatic brain injury. In: Neural Tissue Mechanics, Series: Studies in Mechanobiology, Tissue Engineering and Biomaterials, Editor: Bilston LE, Series Editor: Gefen A, Springer-Verlag

[CR232] Van Essen DC (1997). A tension-based theory of morphogenesis and compact wiring in the central nervous system. Nature.

[CR233] Vappou J, Breton E, Choquet P, Goetz C, Willinger R, Constantinesco A (2007). Magnetic resonance elastography compared with rotational rheometry for in vitro brain tissue viscoelasticity measurement. Magn Reson Mater Phys.

[CR234] Velardi F, Fraternali F, Angelillo M (2006) Anisotropic constitutive equations and experimental tensile behavior of brain tissue. Biomech Model Mechanobiol 5:53–61 10.1007/s10237-005-0007-9 16315049

[CR235] Volman V, Ng LG (2013). Computer modeling of mild axonal injury: implications for axonal signal transmission. Neural Comput.

[CR236] Wall SA, Thomas GP, Johnson D, Byren JC, Jayamohan J, Magdum SA, McAuley DJ, Richards PG (2014). The preoperative incidence of raised intracranial pressure in nonsyndromic sagittal craniosynostosis is underestimated in the literature. J Neurosurg Pediatr.

[CR237] Wang JA, Lin W, Morris T, Banderali U, Juranka PF, Morris CE (2009). Membrane trauma and $$Na^+$$ leak from Nav1.6 channels. Am J Physiol Cell Physiol.

[CR238] Walberer M, Ritschel N, Nedelmann M, Volk K, Mueller C, Tschernatsch M, Stolz E, Blaes F, Bachmann G, Gerriets T (2008). Aggravation of infarct formation by brain swelling in a large territorial stroke: a target for neuroprotection?. J Neurosurg.

[CR239] Watton PN, Raberger NB, Holzapfel GA, Ventikos Y (2009). Coupling the hemodynamic environment to the evolution of cerebral aneurysms: computational framework and numerical examples. J Biomech Eng.

[CR240] Waxman SG, Brill MH (1978). Conduction through demyelinated plaques in multiple sclerosis: computer simulations of facilitation by short internodes. J Neurol Neurosurg Psychiatry.

[CR241] Waxweiler R, Thurman D, Sniezek H, Sosin D, ONeil J (1995). Monitoring the impact of traumatic brain injury: a review and update. J Neurotrauma.

[CR242] Welker W, Jones EG, Peters A (1990). Why does cerebral cortex fissure and fold? A review of determinants of gyri and sulci. Cereb Cortex.

[CR243] Wilkie KP, Drapaca CS, Sivaloganathan S (2012). Aging impact on brain biomechanics with applications to hydrocephalus. Math Med Biol.

[CR244] Wirth B, Sobey I (2006). An axisymmetric and fully 3d poroelastic model for the evolution of hydrocephalus. Math Med Biol.

[CR245] Wirth B, Sobey I (2009). Analytic solution during an infusion test of the linear unsteady poroelastic equations in a spherically symmetric model of the brain. Math Med Biol.

[CR246] Woods RH, Ul-Haq E, Wilkie AO, Jayamohan J, Richards PG, Johnson D, Lester T, Wall SA (2009) Reoperation for intracranial hypertension in TWIST1-confirmed Saethre-Chotzen syndrome: a 15-year review. Plast Reconstr Surg 123:1801–1810 10.1097/PRS.0b013e3181a3f391 PMC2719244 19483581

[CR247] Wright RM, Post A, Hoshizaki B, Ramesh KT (2013). A multiscale computational approach to estimating axonal damage under inertial loading of the head. J Neurotrauma.

[CR248] Wygnanski-Jaffe T, Morad Y, Levin AV (2009). Pathology of retinal hemorrhage in abusive head trauma. Forensic Sci Med Pathol.

[CR249] Xu G, Bayly PV, Taber LA (2009). Residual stress in the adult mouse brain. Biomech Model Mechanobiol.

[CR250] Xu G, Knutsen AK, Dikranian K, Kroenke CD, Bayly PV, Taber LA (2010). Axons pull on the brain, but tension does not drive cortical folding. J Biomech Eng.

[CR251] Zilles K, Palomero-Gallagher N, Amunts K (2013). Development of cortical folding during evolution and ontogeny. Trends Neurosci.

